# Publisher Correction: Electrophysiological population dynamics reveal context dependencies during decision making in human frontal cortex

**DOI:** 10.1038/s41467-023-44259-y

**Published:** 2023-12-15

**Authors:** Wan-Yu Shih, Hsiang-Yu Yu, Cheng-Chia Lee, Chien-Chen Chou, Chien Chen, Paul W. Glimcher, Shih-Wei Wu

**Affiliations:** 1 Institute of Neuroscience, College of Life Sciences, National Yang Ming Chiao Tung University, Taipei, Taiwan, ROC; 2 College of Medicine, National Yang Ming Chiao Tung University, Taipei, Taiwan, ROC; 3 Department of Epilepsy, Neurological Institute, Taipei Veterans General Hospital, Taipei, Taiwan, ROC; 4 Brain Research Center, National Yang Ming Chiao Tung University, Taipei, Taiwan, ROC; 5 Department of Neurosurgery, Neurological Institute, Taipei Veterans General Hospital, Taipei, Taiwan, ROC; 6 Neuroscience Institute, NYU Grossman School of Medicine, New York, NY USA; 7 Department of Neuroscience and Physiology, NYU Grossman School of Medicine, New York, NY USA

**Keywords:** Decision, Reward, Motivation

Correction to: *Nature Communications* 10.1038/s41467-023-42092-x, published online 28 November 2023

The original version of this article contained an error in the “Results” section. A sentence incorrectly read ‘At the group level, the mean regression coefficient (across subjects) was significantly different from 0 (two sample t test, *t* = 4.77, *p* < 0.001)’ instead of ‘At the group level, the mean regression coefficient (across subjects) was significantly different from 0 (one-sample *t* test, two tailed, *t* = 4.77, *p* < 0.001).’ The original article has been corrected.

