# Supplementary material for: Electrophysiological population dynamics reveal context dependencies during decision making in human frontal cortex
Source: Nat Commun. 2023 Nov 28;14:7821. doi: 10.1038/s41467-023-42092-x (PMC10684521; doi:10.1038/s41467-023-42092-x)
Supplement: Supplementary file 1 — Supplementary Information [file 41467_2023_42092_MOESM1_ESM.pdf]

Electrophysiological population dynamics reveal context dependencies during decision making in human frontal cortex

Supplementary Material

Supplementary Figure 1

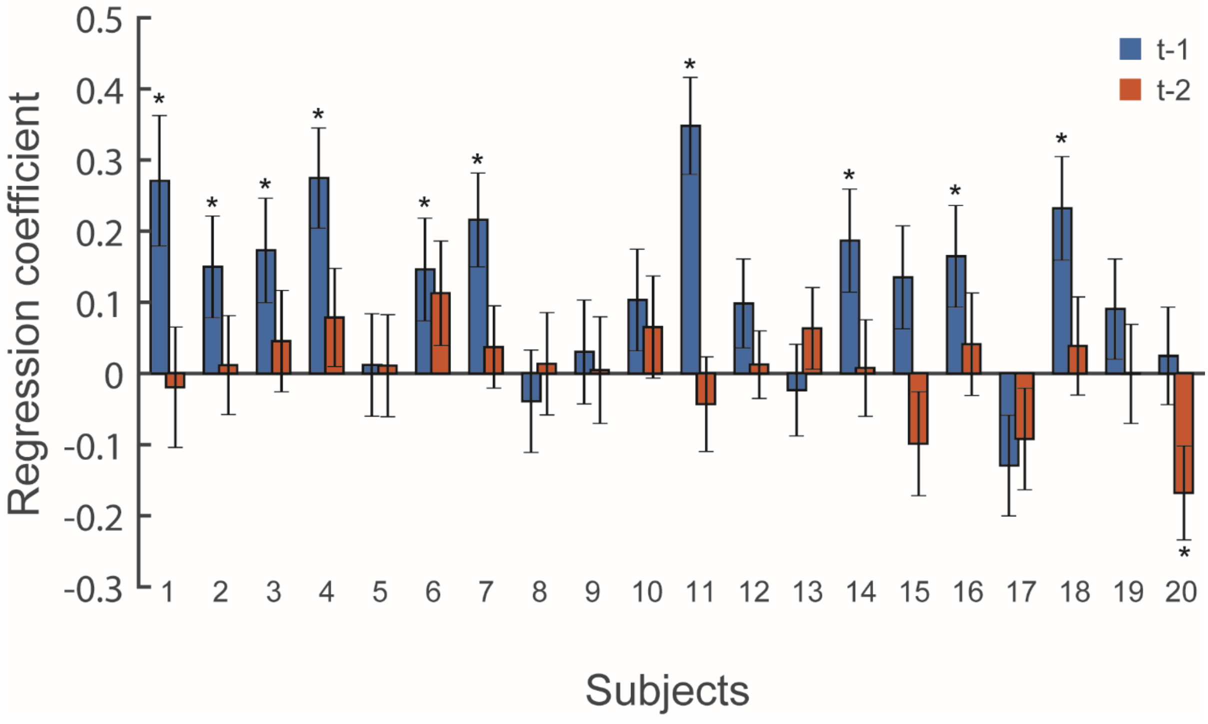

Supplementary Figure 1. Temporal context dependency of subjective value. For each subject, we regressed the subjective value (willingness-to-pay) of the current trial against that of the previous trial (t-1) and two-trials back (t-2). Here we plot the mean regression coefficient (across subjects) of the (t-1) subjective value and (t-2) subjective value. The * symbol indicates $p<0.05$ (one sample t test, two-tailed). Information about sample size (number of trials) for each subject is provided in the Source Data file. The t statistic of these regression coefficients and its corresponding p-value can be found in Supplementary Table 2. Error bars represent ± 1 standard error of the mean. Source data are provided as a Source Data file.

Supplementary Figure 2

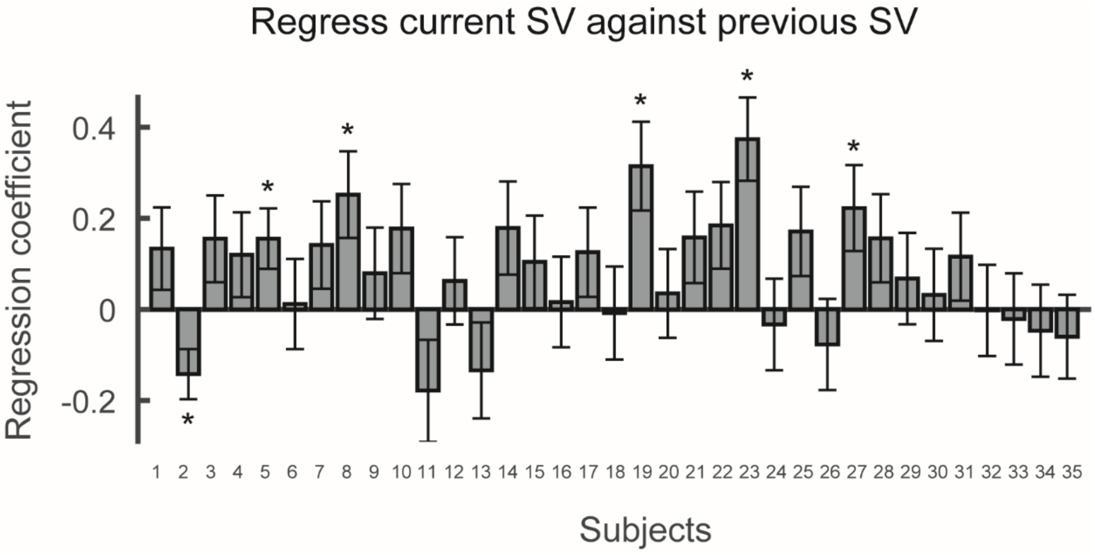

Supplementary Figure 2. Temporal context dependency of subjective value from 35 healthy subjects. For each subject, we regressed the subjective value (willingness-to-pay) of the current trial against that of the previous trial. Here we plot the mean regression coefficient (across subjects) of the previous subjective value. For each subject, we performed a one-sample *t* test ($\alpha = 0.05$, two-tailed) on the regression coefficient. Across all subjects, the *t* statistics ranged from -2.59 to 4.08. Regression coefficients significantly different from 0 at p<0.05 were marked by the * symbol. Information about sample size (number of trials) for each subject is provided in the Source Data file. The *t* statistic of these regression coefficients and its corresponding p-value can be found in Supplementary Table 3. We found that 25 out of 35 subjects showed positive regression coefficient with the previous subjective value. At the group level, the mean regression coefficient (across subjects) was significantly different from 0 (*n*=20, one-sample *t* test, two-tailed, $t = 3.81,\ p < 0.001$). Error bars represent ± 1 standard error of the mean. Source data are provided as a Source Data file.

Supplementary Figure 3

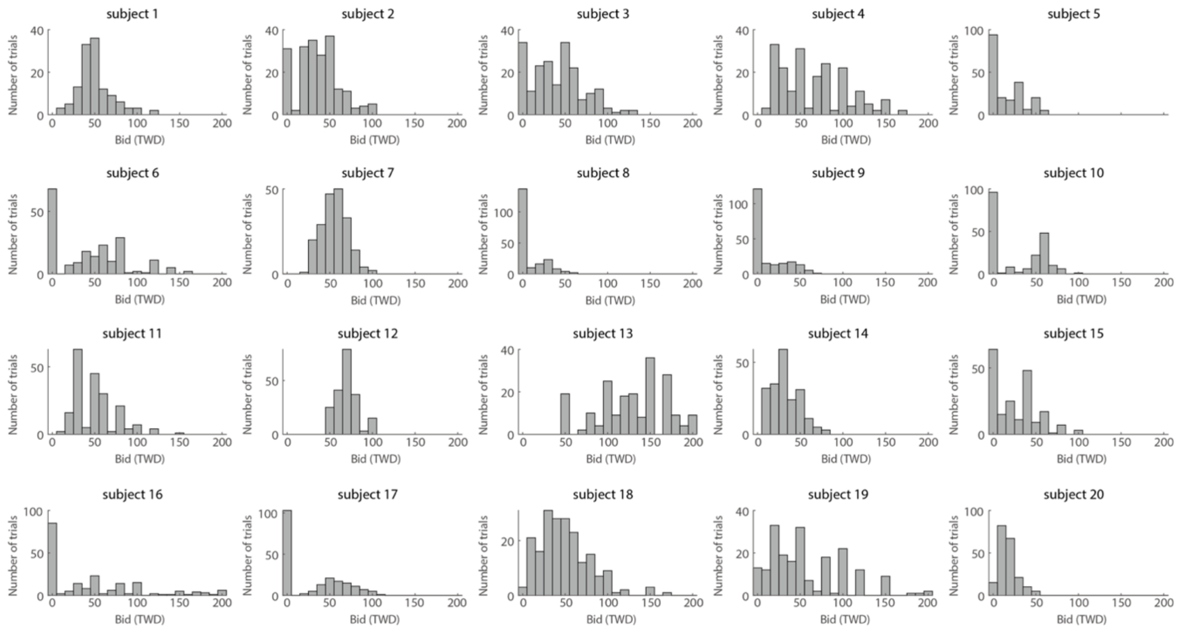

Supplementary Figure 3. Distribution of individual subjects' willingness-to-pay. Source data are provided as a Source Data file.

Supplementary Figure 4

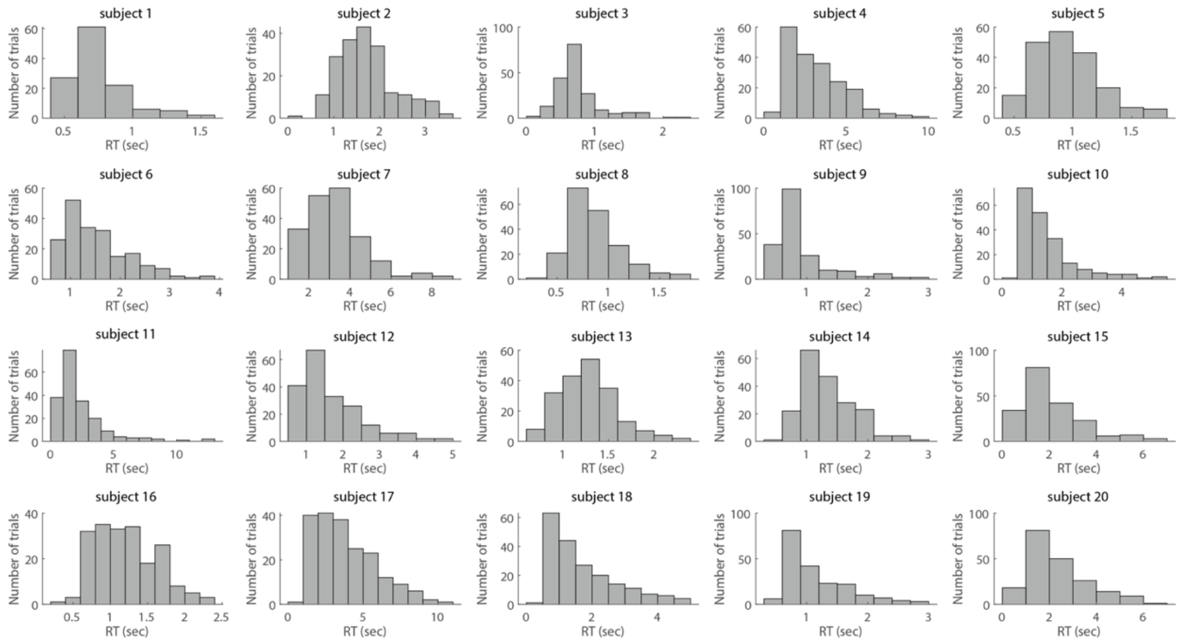

Supplementary Figure 4. Response time (RT) distribution of individual subjects. Source data are provided as a Source Data file.

Supplementary Figure 5

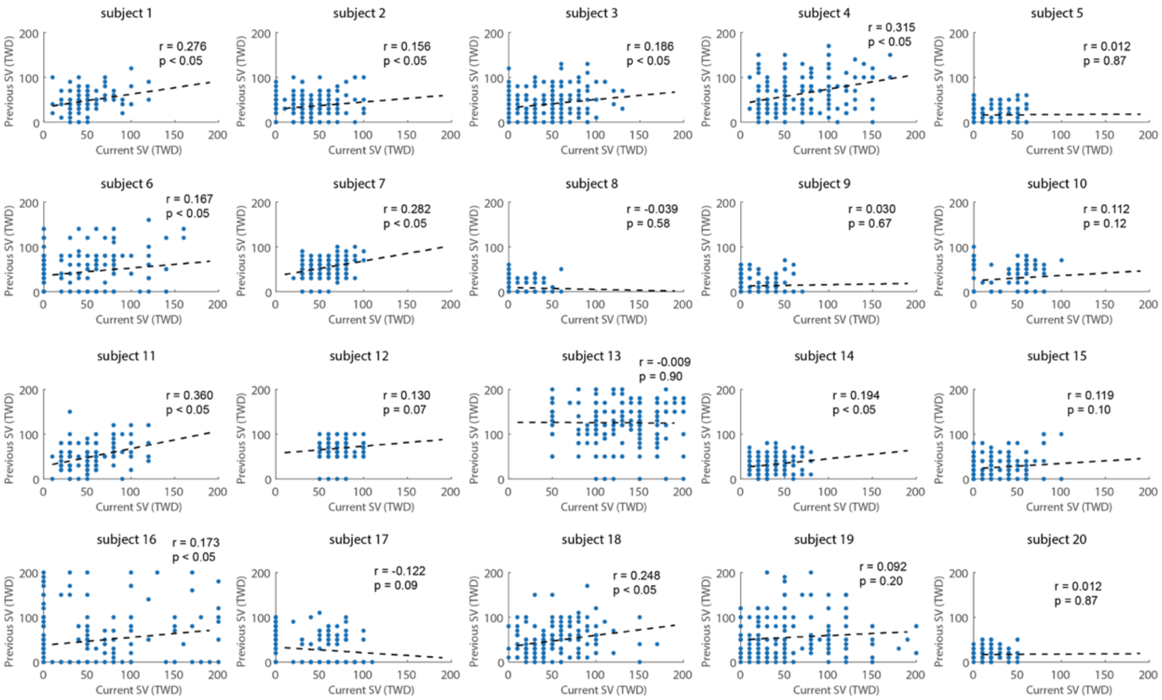

Supplementary Figure 5. Relation between the subjective value of the current trial and the subjective value of the previous trial. For each subject separately, we plot the willingness-to-pay of the previous trial against that of the current trial. *r* indicates the Pearson correlation coefficient, and *p* indicates the corresponding *p*-value (two-tailed test). Source data are provided as a Source Data file.

Supplementary Figure 6

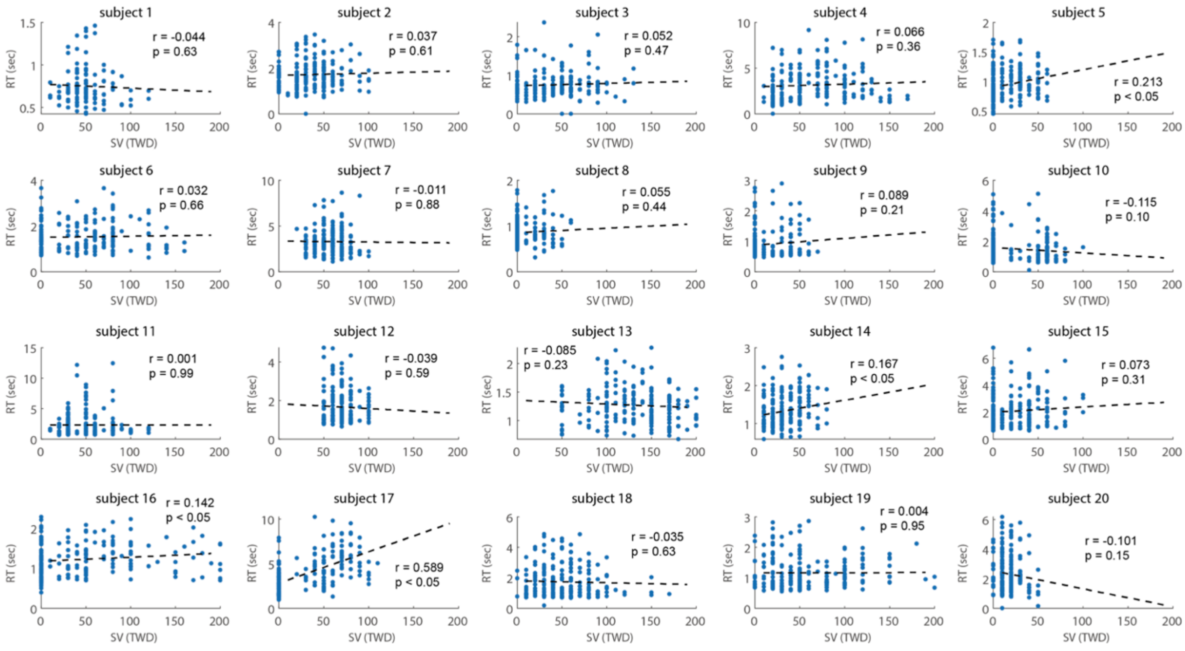

Supplementary Figure 6. Relation between the subjective value and the response time (RT). For each subject separately, we plot the RT against the willingness-to-pay (SV). *r* indicates the Pearson correlation coefficient, and *p* indicates the *p*-value (two-tailed test). Source data are provided as a Source Data file.

Supplementary Figure 7

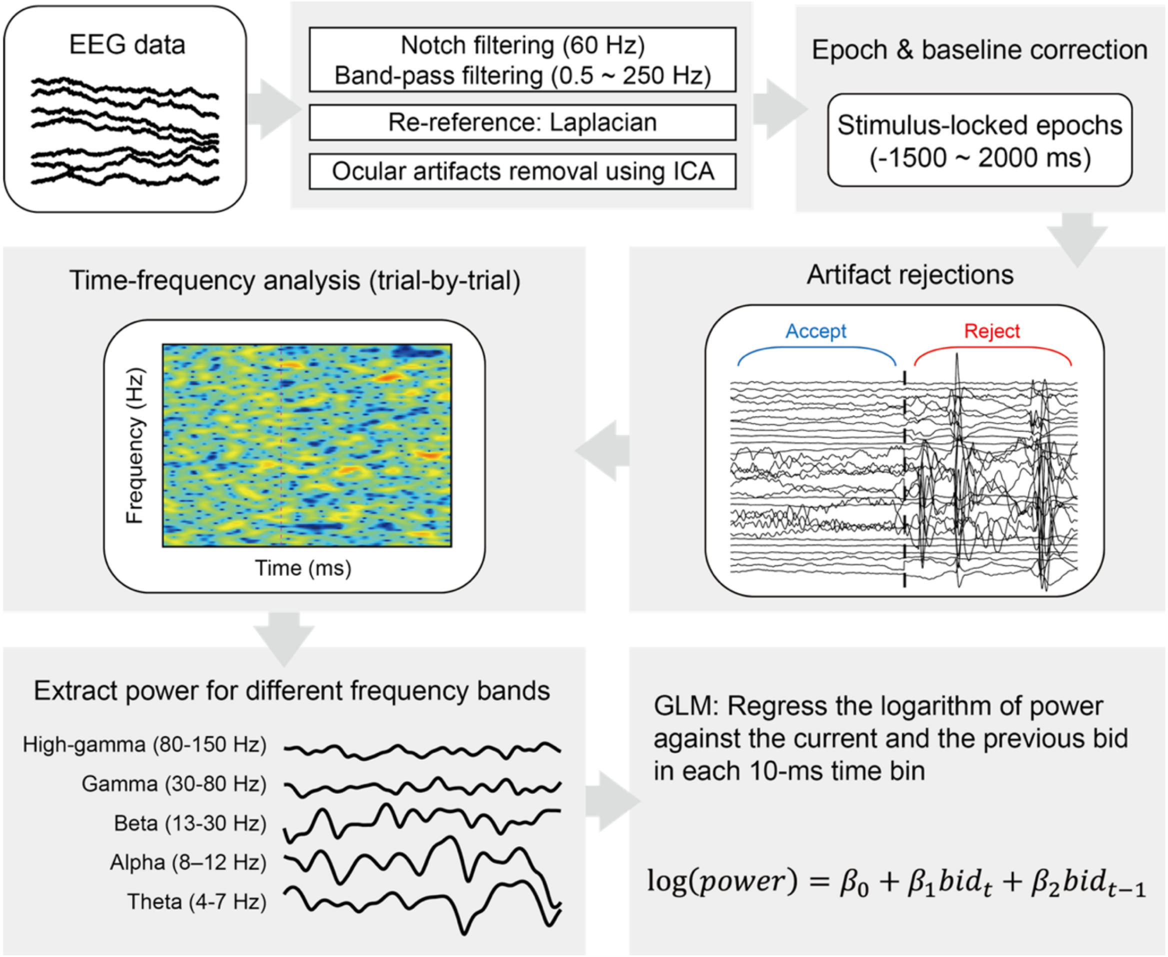

Supplementary Figure 7. Overview of the sEEG data analysis pipeline. First, the sEEG data were filtered to remove uninteresting frequency bands and power-line interference. Second, the sEEG data of each electrode contact were re-referenced to the average of the two neighboring contacts (Laplacian reference). Third, the ocular artifacts were identified and removed from the sEEG data using the Independent Component Analysis (ICA). The trial epoch for each trial was 3.5 s, from 1.5 s before the onset of stimulus to 2 s after stimulus onset. The baseline used for baseline correction was the 2-s pre-stimulus interval. The epochs with interictal activities were identified and excluded from further analysis. After preprocessing, a time-frequency analysis was performed for each epoch. The time series of the power from different frequency bands were then extracted. For each contact, we regressed the logarithm of the power in each 10 ms time bin against the regressors — the bid (willingness-to-pay) subjects revealed in the current trial (bid_t) and that in the previous trial (bid_{t-1}). The estimated regression coefficients (β_1, β_2) reflect the strength and direction of the representations for the subjective value of the current trial (β_1) and that of the previous trial (β_2).

Supplementary Figure 8

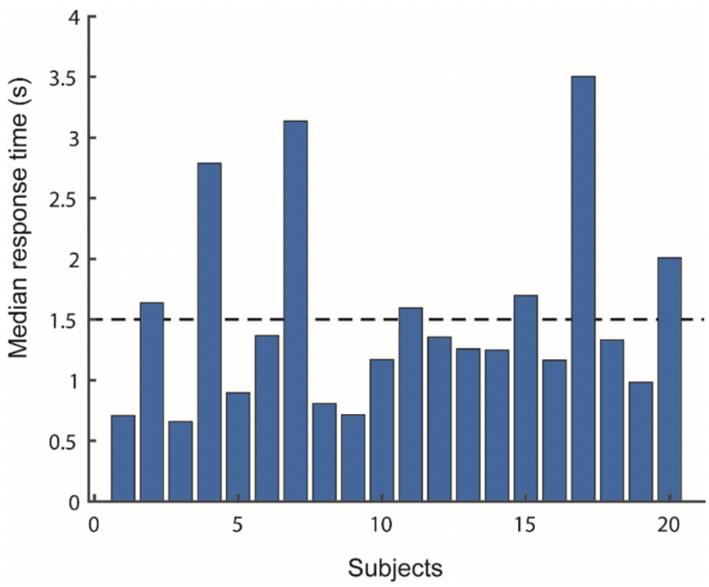

Supplementary Figure 8. Median response time of all subjects. For each subject, we plot his or her median response time. The dashed line indicates the median response time averaged across subjects. Source data are provided as a Source Data file.

Supplementary Figure 9

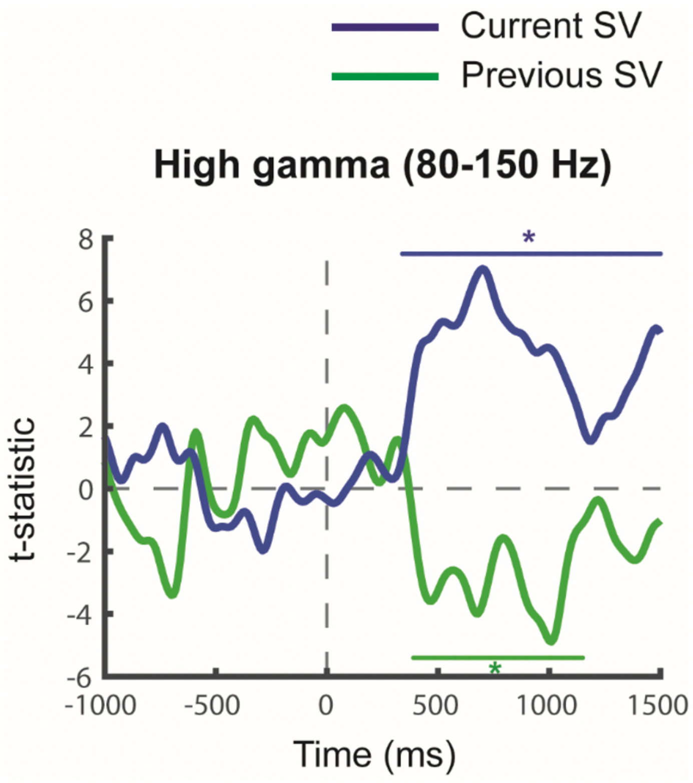

Supplementary Figure 9. Orbitofrontal cortex (OFC) represents subjective value and temporal context. This figure is the *t*-statistic version of Fig. 2b in the main text. Here we plot the *t* statistics of the regression coefficient of the current subjective value (blue) and the previous subjective value (green) based on electrode contacts in the OFC (n=166). Colored (blue or green) horizontal lines with the * symbol on top or beneath indicate the time points with $p<0.05$ (familywise error corrected) using permutation test (two-tailed) with the threshold-free-cluster-enhancement (TFCE) statistic as the test statistic. Source data are provided as a Source Data file.

Supplementary Figure 10

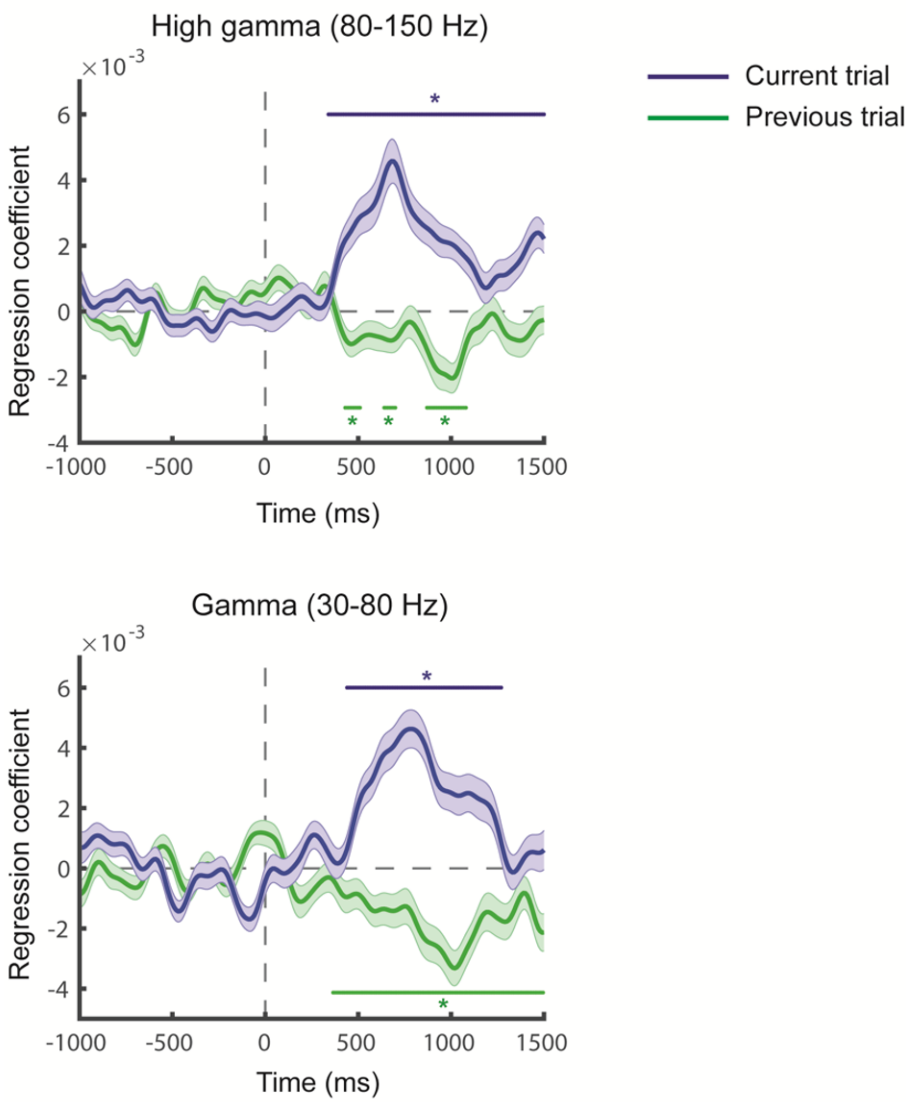

Supplementary Figure 10. Testing the robustness of subjective-value representations in the OFC. Here we reversed the order of steps in GLM-2. Top graph: high-gamma activity. Bottom graph: gamma activity. Results were similar to GLM-2 (Fig. 3b in the main text). Conventions are the same as described in Fig. 3 in the main text. Colored (blue or green) horizontal lines with the * symbol on top or beneath indicate the time points with $p<0.05$ (familywise error corrected) using permutation test (two-tailed) with the threshold-free-cluster-enhancement (TFCE) statistic as the test statistic. Error bands represent ± 1 standard error of the mean. Source data are provided as a Source Data file.

Supplementary Figure 11

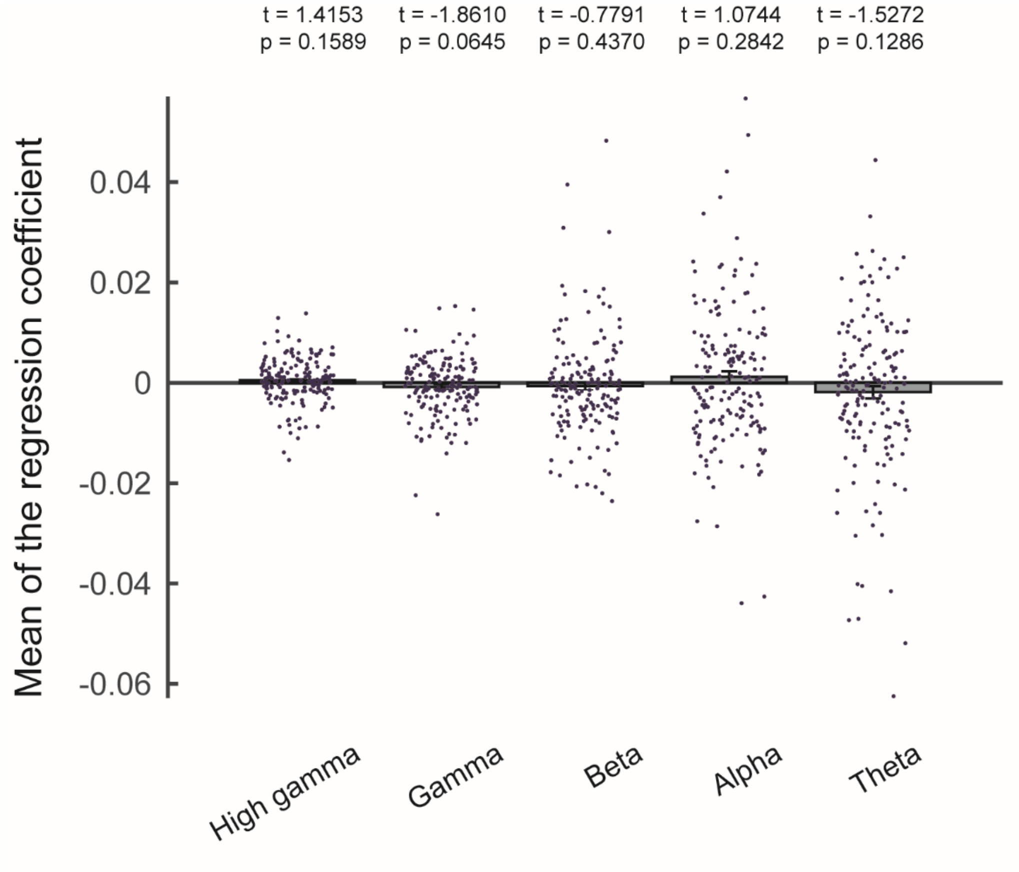

Supplementary Figure 11. Pre-stimulus baseline did not correlate with previous subjective value. In order to examine whether our particular baseline correction method impacted the previous-value results in the OFC (Figs. 2 and 3 in the main text), we performed a linear regression analysis where we regressed baseline activity – the average power of pre-stimulus period – against previous subjective value. Here we plot the regression coefficient of previous subjective value at different frequency bands. We did not find the regression coefficients to be statistically significant from 0 (n=166, t test, two-tailed, p>0.05), suggesting that the previous-value results were not driven by the baseline correction method we employed. Each data point at each frequency band represents a single electrode contact in the OFC (166 OFC contacts). Error bars represent ± 1 standard error of the mean. Source data are provided as a Source Data file.

Supplementary Figure 12

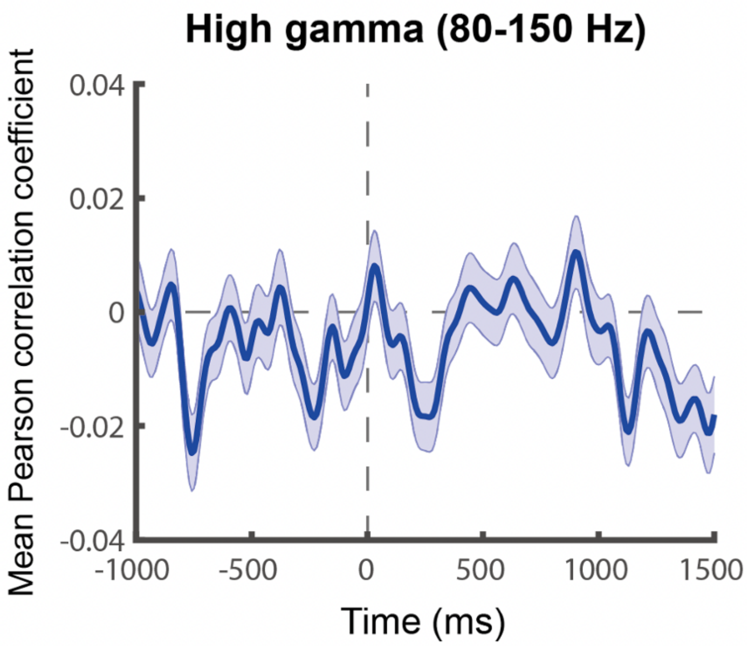

Supplementary Figure 12. Signal autocorrelation. Here we plot the mean Pearson correlation (averaged across subjects) in the high-gamma power between the current trial and the previous trial. Error bands represent ± 1 standard error of the mean. Source data are provided as a Source Data file.

Supplementary Figure 13

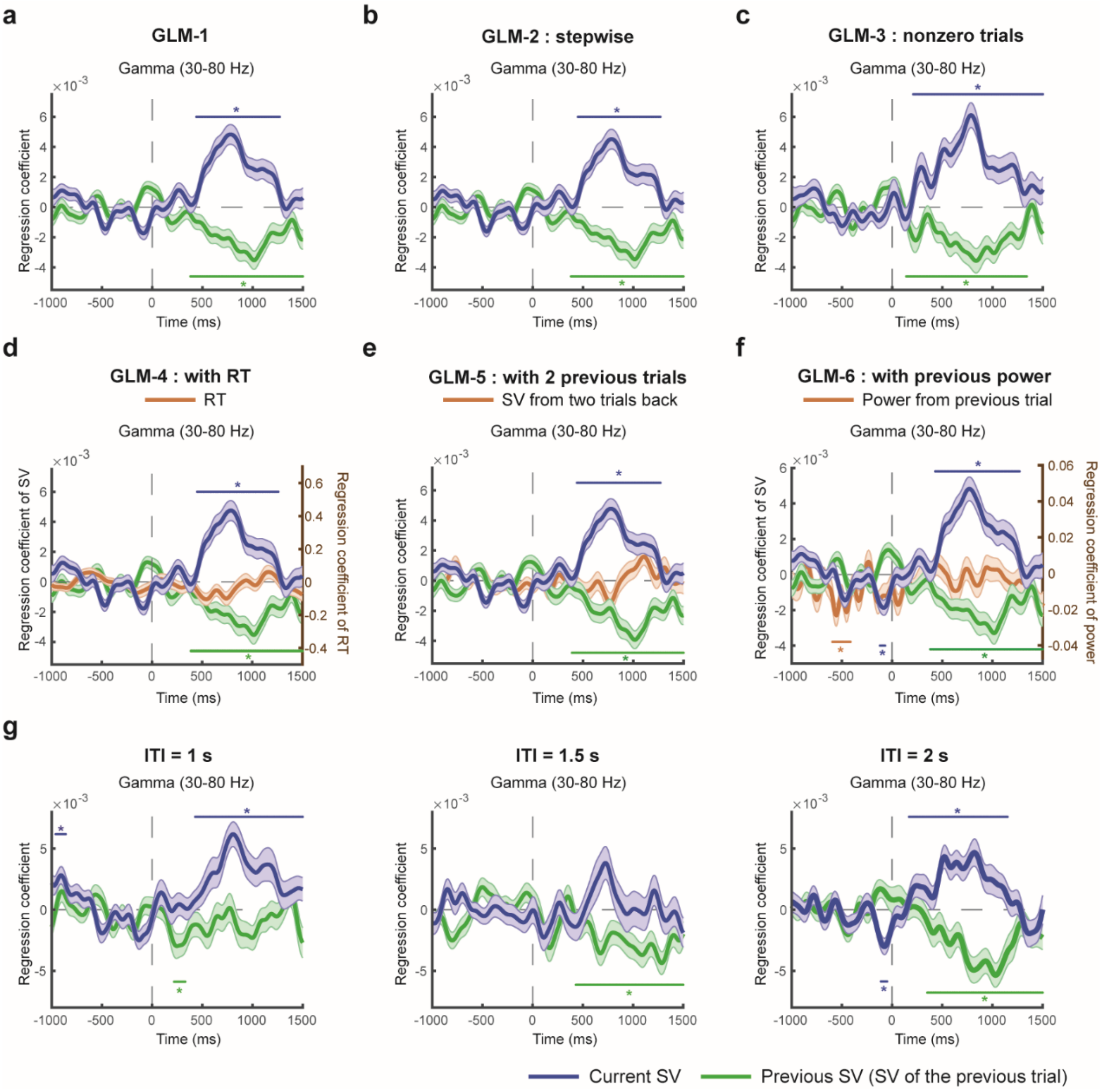

Supplementary Figure 13. Testing the robustness of subjective-value representations in the OFC. This figure is the gamma activity (30-80 Hz) version of Fig. 3 in the main text. **a.** GLM-1. **b.** GLM-2. **c.** GLM-3. **d.** GLM-4. **e.** GLM-5. **f.** GLM-6. **g.** Evaluating GLM-1 at different inter-trial intervals (ITIs). We sorted trials according to the preceding ITI (1 s, 1.5 s, or 2 s) and estimated GLM-1 separately for each possible ITI. Colored (blue or green) horizontal lines with the * symbol on top or beneath indicate the time points with $p<0.05$ (familywise error corrected) using permutation test (two-tailed) with the threshold-free-cluster-enhancement (TFCE) statistic as the test statistic. Error bands represent ± 1 standard error of the mean. Source data are provided as a Source Data file.

Supplementary Figure 14

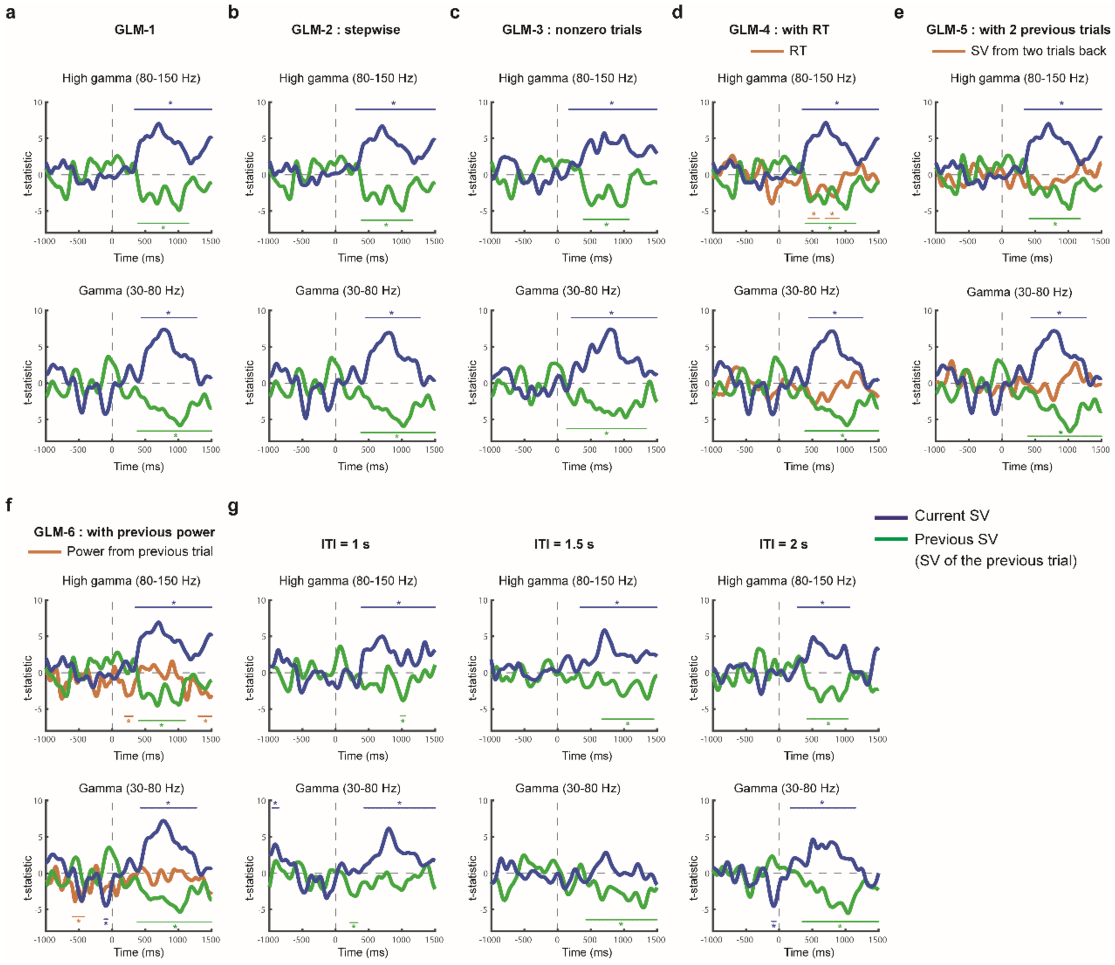

Supplementary Figure 14. This figure is the *t*-statistic version of Fig. 3 in the main text and Supplementary Fig. 13. **a.** GLM-1. **b.** GLM-2. **c.** GLM-3. **d.** GLM-4. **e.** GLM-5. **f.** GLM-6. **g.** Evaluating GLM-1 at different inter-trial intervals (ITIs). Top row: high-gamma activity (the *t*-statistic version of Fig. 3); bottom row: gamma activity (the *t*-statistic version of Supplementary Fig. 13). Colored (blue or green) horizontal lines with the * symbol on top or beneath indicate the time points with $p<0.05$ (familywise error corrected) using permutation test (two-tailed) with the threshold-free-cluster-enhancement (TFCE) statistic as the test statistic. Source data are provided as a Source Data file.

Supplementary Figure 15

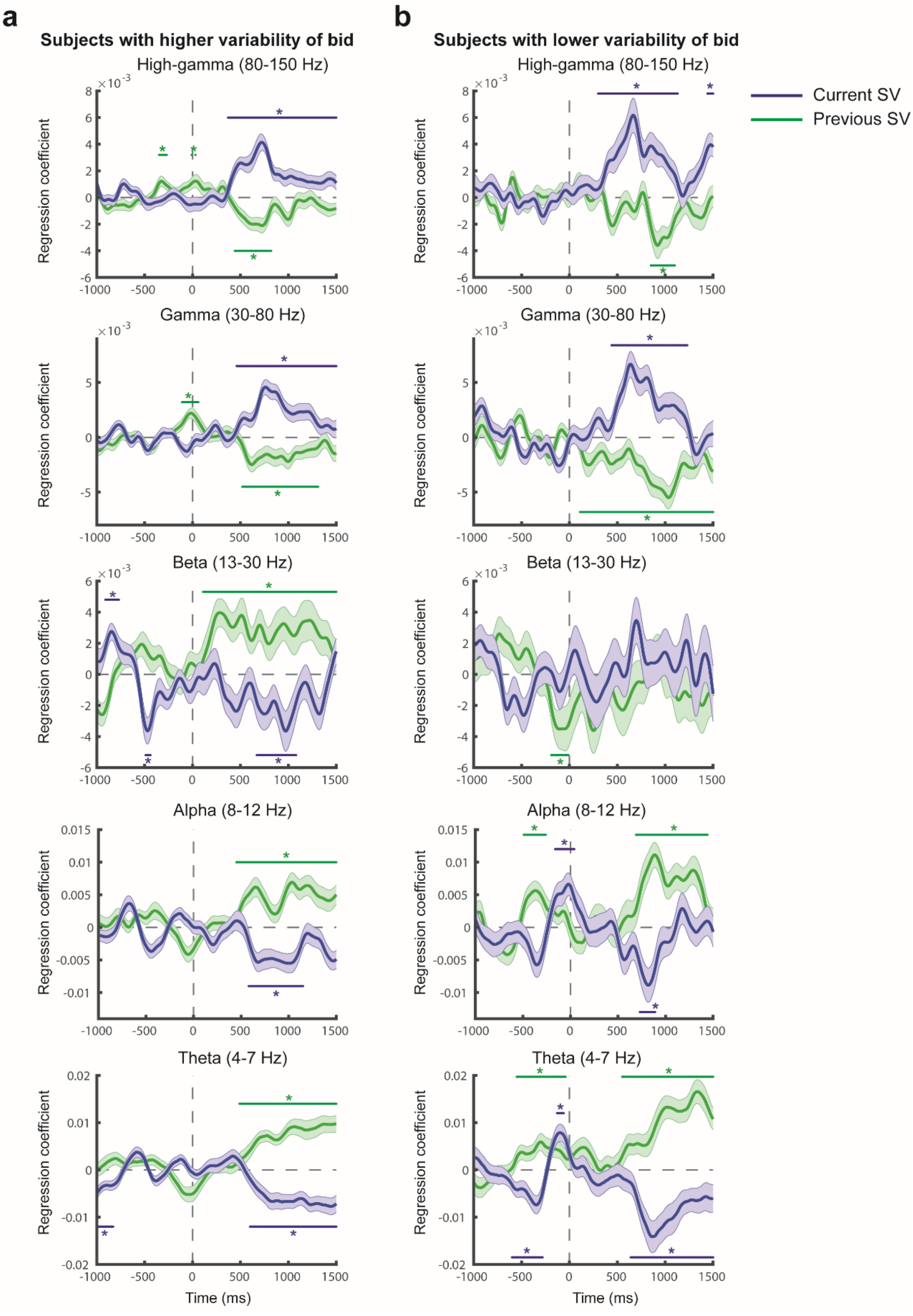

Supplementary Figure 15. Subjective-value representations in the OFC for subjects with

different variability of subjective value. We split the subjects into two different groups according to the variability of their subjective value (willingness-to-pay). **a.** The 10 subjects with larger variability of subjective value (94 contacts). **b.** The 10 subjects with lower variability of subjective value (72 contacts). Conventions are the same as in Figs. 3 and 6 in the main text. Colored (blue or green) horizontal lines with the * symbol on top or beneath indicate the time points with $p<0.05$ (familywise error corrected) using permutation test (two-tailed) with the threshold-free-cluster-enhancement (TFCE) statistic as the test statistic. Error bands represent ± 1 standard error of the mean. Source data are provided as a Source Data file.

Supplementary Figure 16

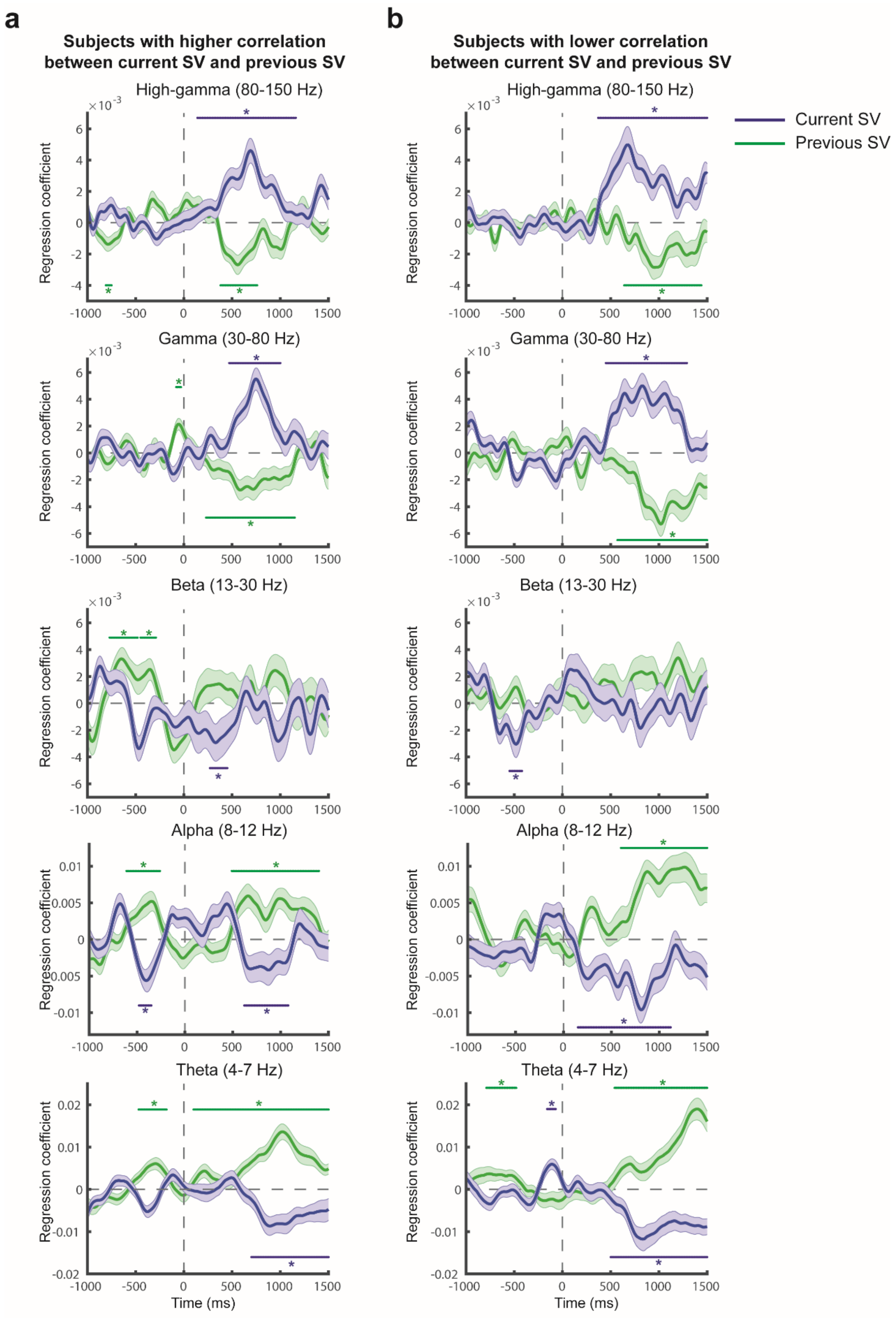

Supplementary Figure 16. Subjective-value representations in the OFC for subjects with

different degree of correlations between the current subjective value and the previous subjective value. We split the subjects into two groups according to the degree of correlation. **a.** The 10 subjects with larger correlation between the current subjective value and the previous subjective value (87 contacts). **b.** The 10 subjects with smaller correlation between current subjective value and the previous subjective value (79 contacts). Conventions are the same as described in Figs. 3 and 6 in the main text. Colored (blue or green) horizontal lines with the * symbol on top or beneath indicate the time points with $p<0.05$ (familywise error corrected) using permutation test (two-tailed) with the threshold-free-cluster-enhancement (TFCE) statistic as the test statistic. Error bands represent ± 1 standard error of the mean. Source data are provided as a Source Data file.

Supplementary Figure 17

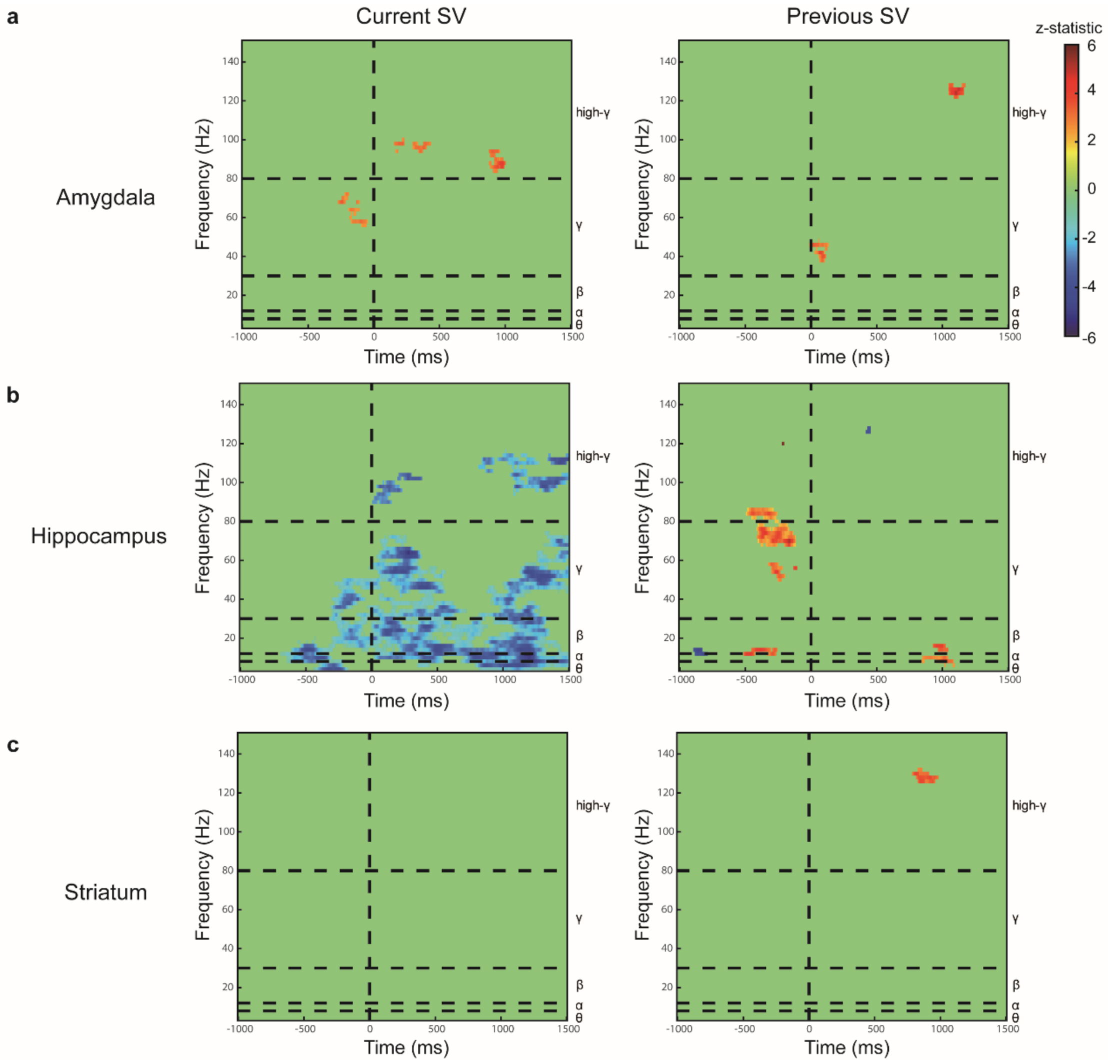

Supplementary Figure 17. Time-frequency representations for subjective value in subcortical regions. **a.** Amygdala. **b.** Hippocampus. **c.** Striatum. For each brain region, the heatmap plots the *z* statistic of the regression coefficient for the current subjective value (left graph) and previous subjective value (right graph). Significant clusters in these two-dimensional maps were identified by contiguous points in the time-frequency space that survived multiple testing with a familywise error rate of 0.05 according to permutation test (500 permutations; two-tailed) with threshold-free-cluster-enhancement (TFCE) as the test statistic (Orange: significant positive correlation; blue: significant negative correlation; green: non-significant results). Source data are provided as a Source Data file.

Supplementary Figure 18

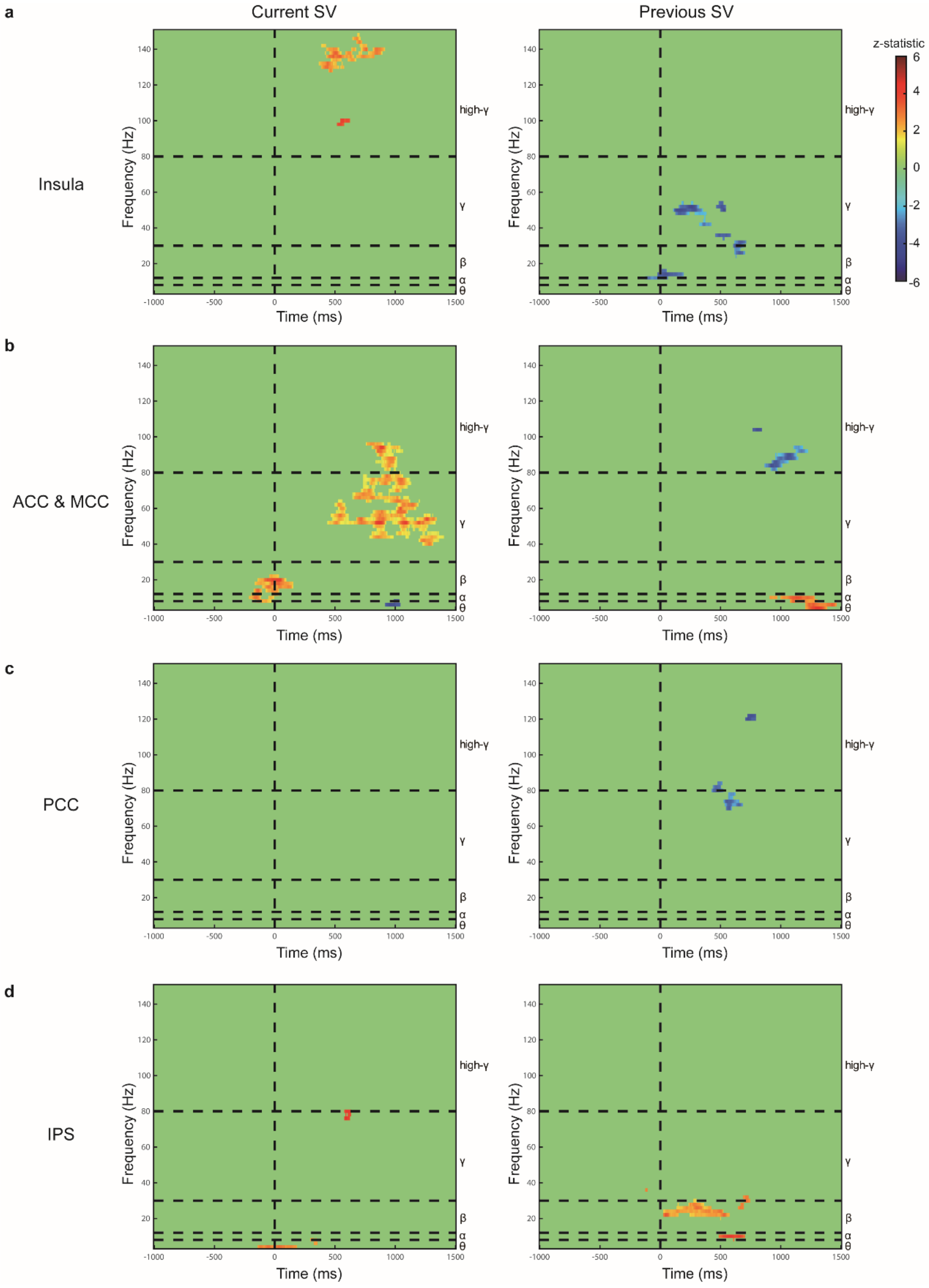

Supplementary Figure 18. Time-frequency representations for subjective value in cortical regions. **a.** Insula. **b.** Anterior and midcingulate cortex (ACC and MCC). **c.** Posterior cingulate cortex (PCC). **d.** Intraparietal sulcus (IPS). For each brain region, the heatmap plots

the *z* statistic of the regression coefficient for the current subjective value (left graph) and previous subjective value (right graph). Significant clusters in these two-dimensional maps were identified by contiguous points in the time-frequency space that survived multiple testing with a familywise error rate of 0.05 according to permutation test (500 permutations; two-tailed) with threshold-free-cluster-enhancement (TFCE) as the test statistic (Orange: significant positive correlation; blue: significant negative correlation; green: non-significant results). Source data are provided as a Source Data file.

Supplementary Figure 19

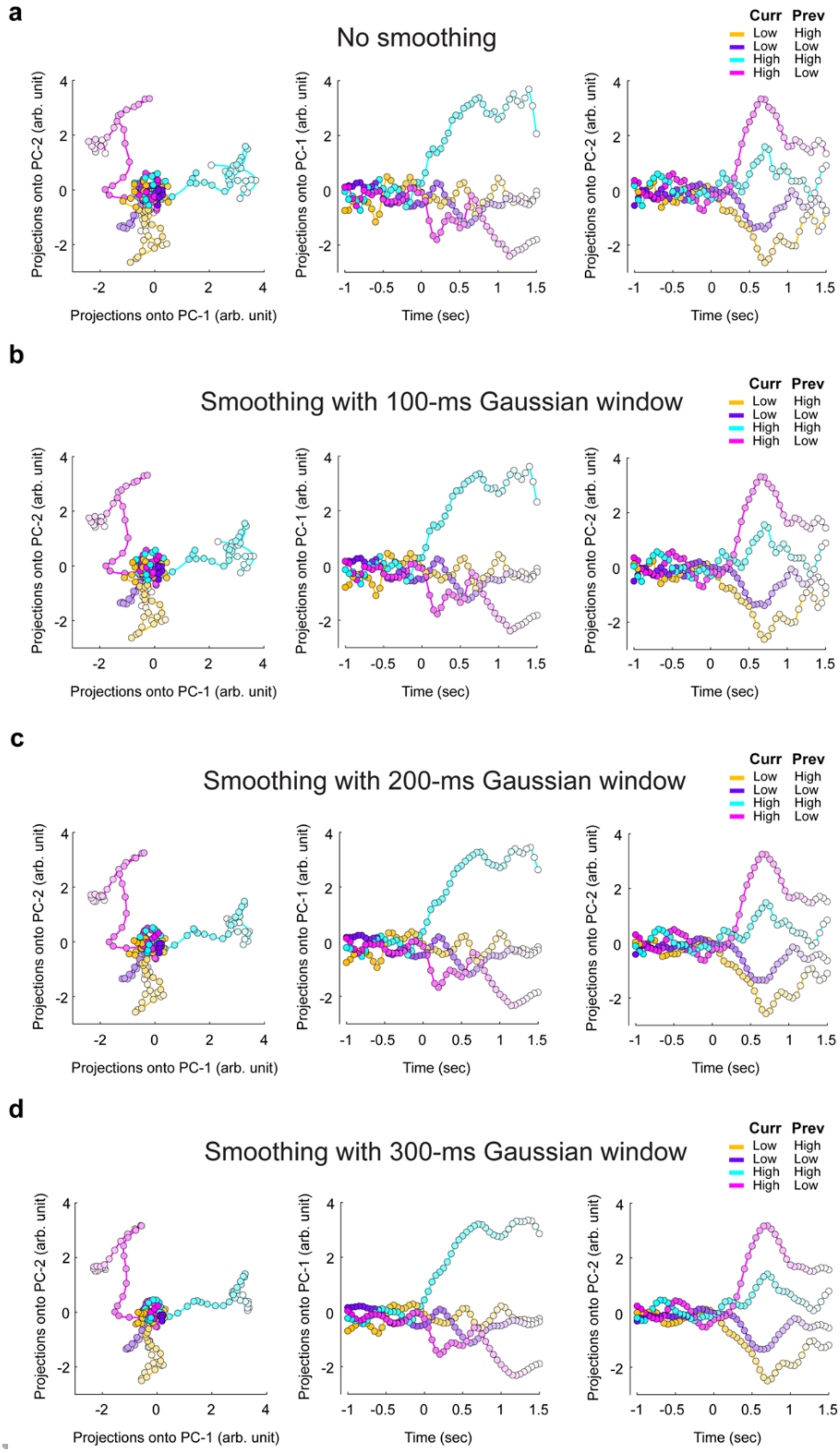

Supplementary Figure 19. Trajectories of OFC population high-gamma activity in the space of the first and second principal components (PC-1, PC-2) under different smoothing parameters. **a.** No smoothing was applied to the trial-level high-gamma timeseries data. **b.** Data were smoothed with a 100-ms Gaussian window. **c.** Data were smoothed with a 200-ms Gaussian window. **d.** Data were smoothed with a 300-ms Gaussian window. Source data are provided as a Source Data file.

Supplementary Figure 20

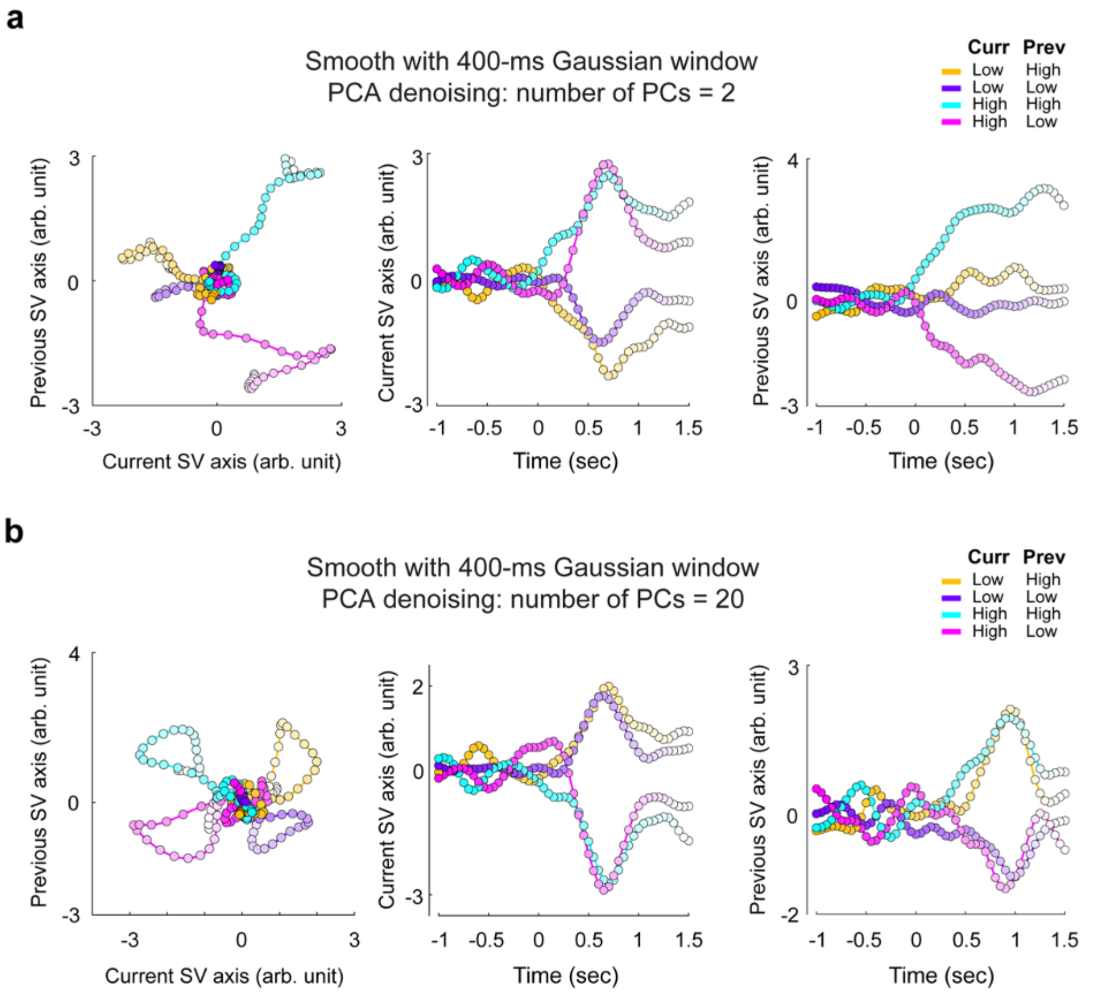

Supplementary Figure 20. Regression subspace analysis. Trajectories of OFC population high-gamma activity in the space of current subjective value (Current SV axis) and previous subjective value (Previous SV axis) under different PCA-based denoising setup when the trial-level high-gamma timeseries data were smoothed with a 400-ms Gaussian time window. **a.** When the number of PCs included is 2. **b.** When the number of PCs included is 20. Source data are provided as a Source Data file.

Supplementary Figure 21

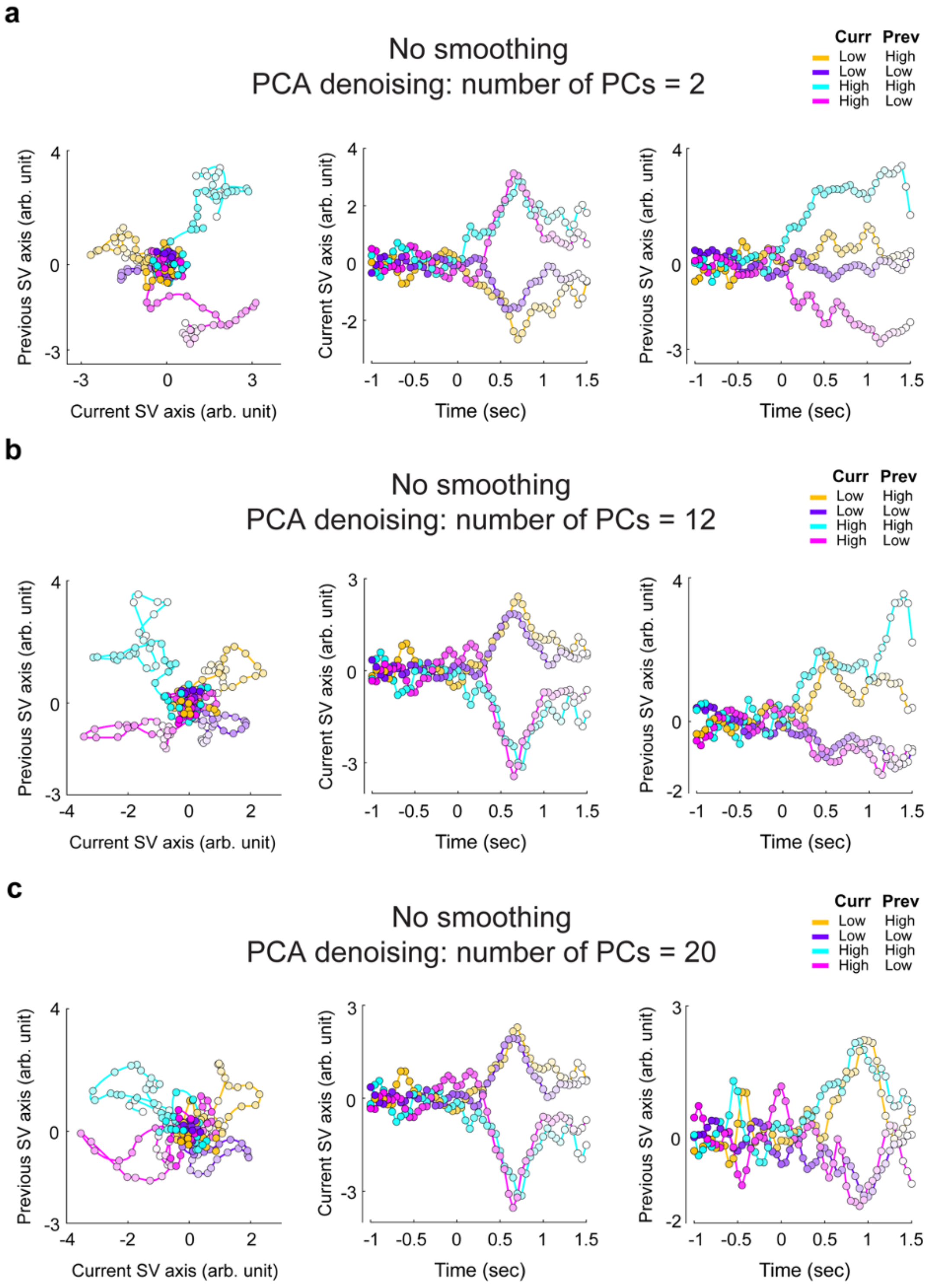

Supplementary Figure 21. Regression subspace analysis. Trajectories of OFC population high-gamma activity in the space of current subjective value (Current SV axis) and previous subjective value (Previous SV axis) under different PCA-based denoising setup when no smoothing was applied to the trial-level high-gamma timeseries data. **a.** The number of PCs

included in the denoising matrix is 2. **b.** 12 PCs were included in the denoising matrix. **c.** 20 PCs were included in the denoising matrix. Source data are provided as a Source Data file.

Supplementary Figure 22

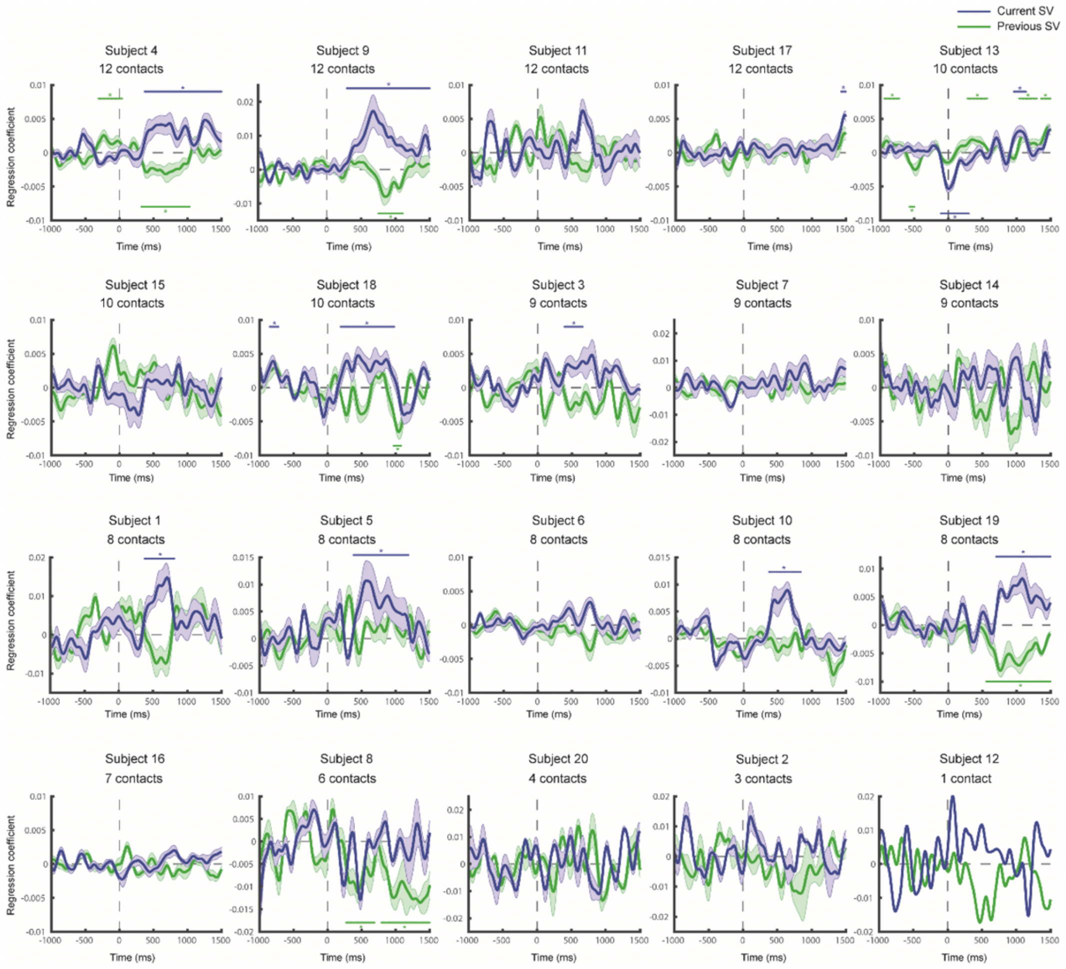

Supplementary Figure 22. Individual subjects' results on subjective value and temporal context in the OFC. Here we plot the mean regression coefficient of the current subjective value (blue) and the previous subjective value (green) for each subject separately. Information about the number of electrode contacts for each subject is also provided. Error bands represent ±1 standard error of the mean. The * symbol indicates statistically significant result at $p<0.05$, familywise error corrected based on permutation test (two-tailed) using TFCE as the test statistic. Error bands represent ±1 standard error of the mean. Source data are provided as a Source Data file.

Supplementary Figure 23

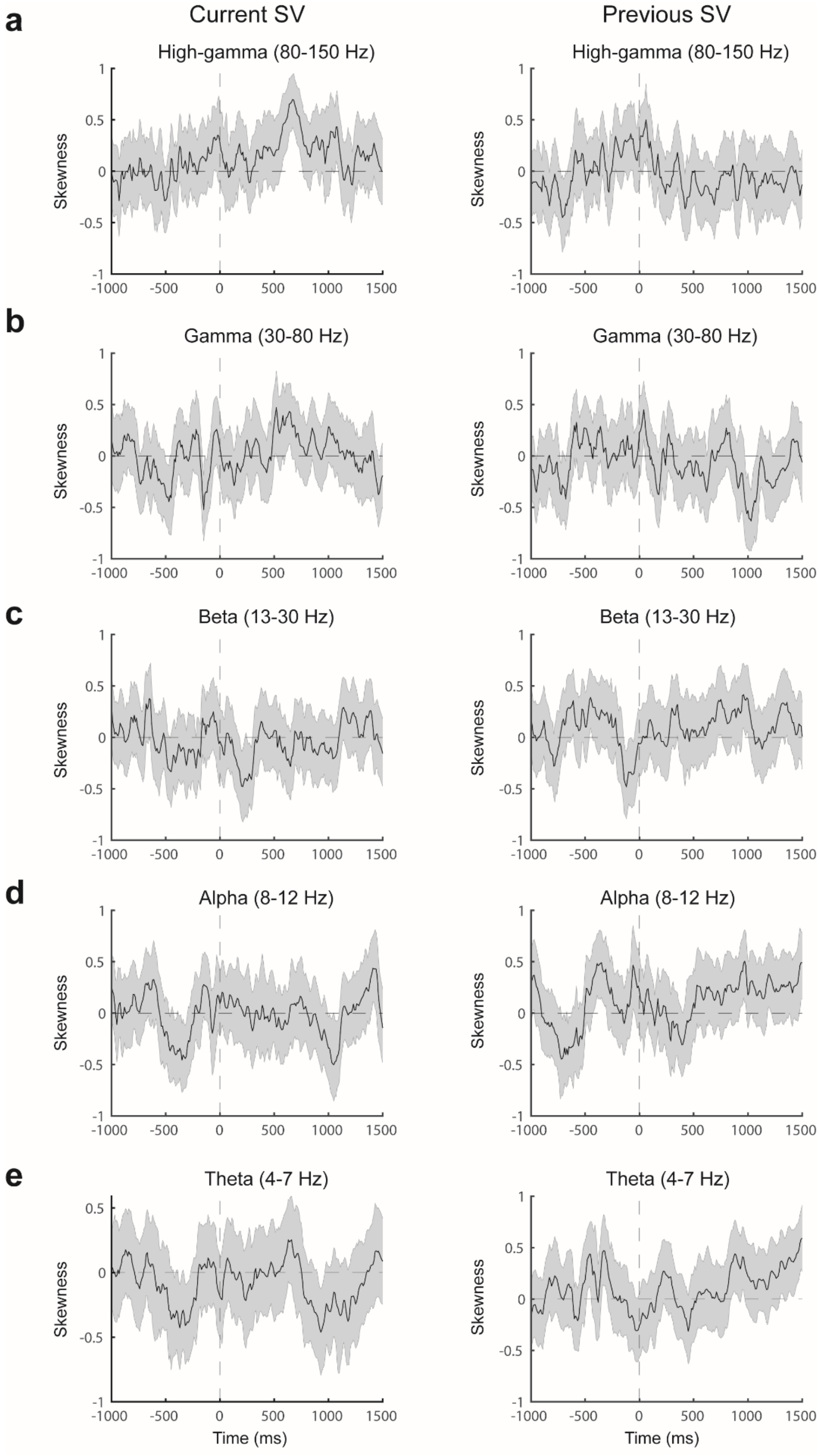

Supplementary Figure 23. Sample skewness of the regression coefficients in GLM-1 (Current SV, Previous SV) from the OFC contacts. **a.** Data were High-gamma power (80-150 Hz). **b.** Data were Gamma power (30-80 Hz). **c.** Data were Beta power (13-30 Hz). **d.** Data were Alpha power (8-12 Hz). **e.** Data were Theta power (4-7 Hz). Error bands cover the 95% confidence interval. Source data are provided as a Source Data file.

Supplementary Figure 24

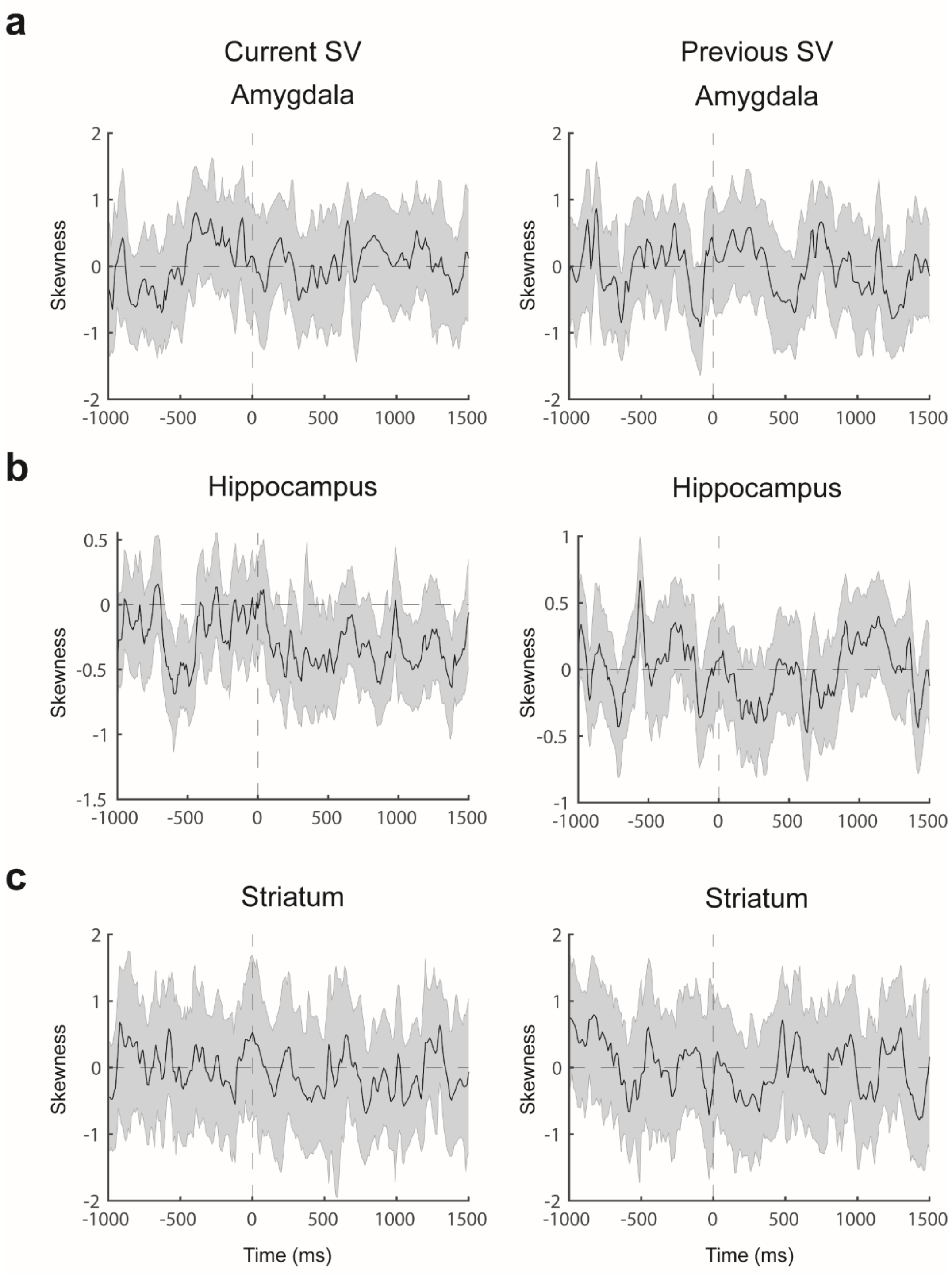

Supplementary Figure 24. Sample skewness of the regression coefficients in GLM-1 (Current SV, Previous SV) from the subcortical contacts. Data were High-gamma power (80-150 Hz). **a.** Amygdala (30 contacts). **b.** Hippocampus (126 contacts). **c.** Striatum (25 contacts). Error bands cover the 95% confidence interval. Source data are provided as a

Source Data file.

Supplementary Figure 25

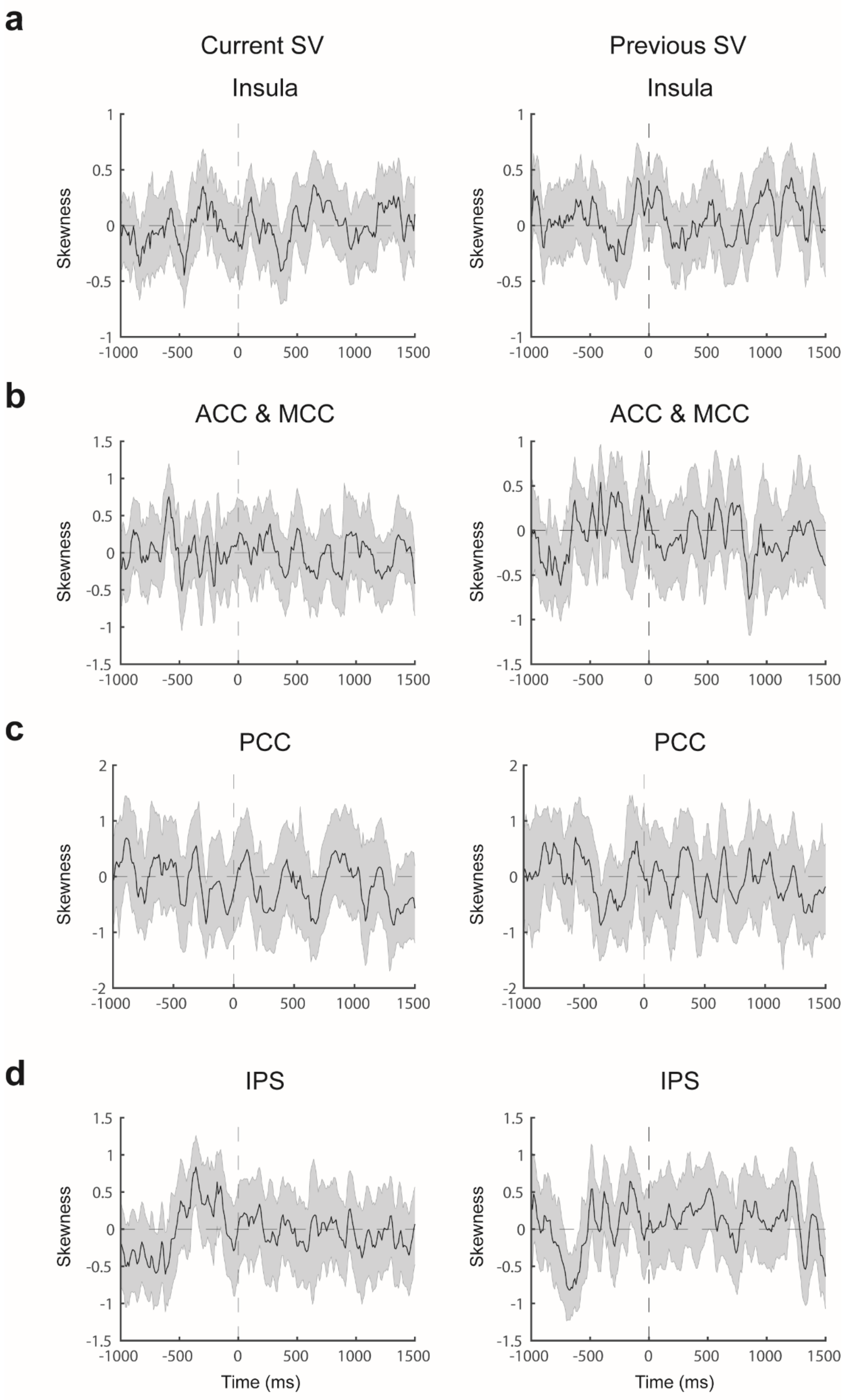

Supplementary Figure 25. Sample skewness of the regression coefficients in GLM-1 (Current SV, Previous SV) from the cortical contacts other than OFC. Data were High-gamma power (80-150 Hz). **a.** Insula (169 contacts). **b.** Anterior cingulate and midcingulate cortex (ACC and MCC, 81 contacts). **c.** Posterior cingulate cortex (PCC, 31 contacts). **d.** Intraparietal sulcus (IPS, 62 contacts). Error bands cover the 95% confidence interval. Source data are provided as a Source Data file.

Supplementary Table 1: *t* statistic of regression coefficient of the previous SV in a linear regression analysis where the data were the current SV. The *p*-values are based on one sample *t* test (two-tailed). Source data are provided as a Source Data file.

Subject	*t* statistic	*p*-value
1	3.16	0.002
2	2.21	0.028
3	2.63	0.009
4	4.63	<0.001
5	0.17	0.865
6	2.37	0.019
7	4.10	<0.001
8	-0.55	0.583
9	0.42	0.674
10	1.58	0.116
11	5.37	<0.001
12	1.81	0.071
13	-0.12	0.903
14	2.76	0.006
15	1.67	0.097
16	2.45	0.015
17	-1.72	0.087
18	3.57	<0.001
19	1.30	0.195
20	0.17	0.868

Supplementary Table 2: *t* statistic of regression coefficient of the previous trial (*t*-1) SV and two-trial back (*t*-2) SV where the current SV were the data in a linear regression analysis. The *p*-values are based on one-sample *t* test (two-tailed). Source data are provided as a Source Data file.

Subject	Previous trial (t-1)		Two-trials back (t-2)	
	t statistic	*p*-value	*t* statistic	*p*-value
1	2.96	0.004	-0.23	0.820
2	2.10	0.037	0.17	0.866
3	2.36	0.020	0.64	0.525
4	3.91	<0.001	1.14	0.256
5	0.17	0.869	0.15	0.878
6	2.03	0.044	1.54	0.126
7	3.28	0.001	0.64	0.521
8	-0.54	0.588	0.19	0.850
9	0.41	0.679	0.06	0.950
10	1.45	0.150	0.91	0.364
11	5.11	<0.001	-0.65	0.518
12	1.58	0.117	0.26	0.794
13	-0.36	0.717	1.10	0.271
14	2.57	0.011	0.11	0.910
15	1.87	0.064	-1.35	0.178
16	2.30	0.022	0.57	0.569
17	-1.83	0.069	-1.29	0.199
18	3.19	0.002	0.56	0.576
19	1.29	0.199	-0.01	0.994
20	0.36	0.719	-2.54	0.012

Supplementary Table 3: *t* statistic of regression coefficient of the previous SV where the current SV were the data from 35 healthy subjects in a linear regression analysis. The *p*-values are based on one-sample *t* test (two-tailed). Source data are provided as a Source Data file.

Subject	*t* statistic	*p*-value
1	1.47	0.144
2	-2.59	0.011
3	1.63	0.107
4	1.28	0.202
5	2.34	0.020
6	0.12	0.906
7	1.48	0.144
8	2.65	0.009
9	0.79	0.432
10	1.81	0.074
11	-1.60	0.113
12	0.65	0.515
13	-1.27	0.206
14	1.75	0.083
15	1.02	0.308
16	0.16	0.872
17	1.28	0.204
18	-0.08	0.937
19	3.22	0.002
20	0.36	0.721
21	1.57	0.119
22	1.93	0.056
23	4.08	<0.001
24	-0.33	0.742
25	1.74	0.085
26	-0.77	0.443
27	2.35	0.021
28	1.61	0.110
29	0.67	0.502
30	0.31	0.754
31	1.20	0.233
32	-0.02	0.982

33	-0.21	0.832
34	-0.46	0.643
35	-0.65	0.514

Supplementary Table 4: *t* statistic of regression coefficient of response time where the current SV were the data in a linear regression analysis. The *p*-values are based on one - sample *t* test (two-tailed). Source data are provided as a Source Data file.

Subject	*t* statistic	*p*-value
1	-0.49	0.626
2	0.52	0.606
3	0.73	0.467
4	0.92	0.358
5	3.06	0.003
6	0.45	0.655
7	-0.16	0.875
8	0.77	0.443
9	1.25	0.214
10	-1.63	0.105
11	0.01	0.994
12	-0.54	0.588
13	-1.20	0.232
14	2.36	0.020
15	1.02	0.307
16	2.02	0.045
17	10.20	<0.001
18	-0.49	0.625
19	0.06	0.954
20	-1.43	0.154

Supplementary Table 5: OFC high-gamma (80-150 Hz) activity. Summary of statistical analysis using different GLMs to examine the robustness of the effects of current and previous subjective value. Conventions are the same as Table 1 in the main text. The *p*-values correspond to the maximum or minimum TFCE and are based on permutation test (two-tailed) using TFCE as the test statistic. Source data are provided as a Source Data file.

Model	Regressor	Cluster size	Start time (ms)	End time (ms)	Maximum or minimum TFCE	*p*-value	Peak time (ms)
GLM-2	Current SV	120	310	1500	14678969	<0.0001	710
	Previous SV	78	390	1160	-1556068	0.0006	990
GLM-3	Current SV	134	170	1500	12564742	<0.0001	700
	Previous SV	70	390	1080	-1319450	0.0008	490
GLM-4	Current SV	116	350	1500	16198243	<0.0001	700
	Previous SV	76	400	1150	-1589928	0.0004	680
	RT	17	440	600	-399944	0.0277	510
		21	700	900	-423796	0.0248	820
GLM-5	Current SV	117	340	1500	16336977	<0.0001	700
	Previous SV	78	410	1180	-1413531	0.0002	1020
GLM-6	Current SV	116	350	1500	17453375	<0.0001	680
	Previous SV	71	400	1100	-1565380	0.0003	680
	Power from previous trial	13	190	310	-173119	0.0205	230
		21	1300	1500	-304044	0.0034	1470
GLM-1 ITI = 1 s	Current SV	112	390	1500	3322871	0.0001	690
	Previous SV	8	980	1050	-223952	0.038	1010
GLM-1 ITI = 1.5 s	Current SV	117	340	1500	3364757	<0.0001	700
	Previous SV	79	670	1450	-541001	0.0074	980
GLM-1 ITI = 2 s	Current SV	79	280	1060	2505369	<0.0001	530
	Previous SV	63	420	1040	-1230608	0.002	610

Supplementary Table 6: OFC gamma activity (30-80 Hz). Summary of statistical analysis using different GLMs to examine the robustness of the effects of current and previous subjective value. Conventions are the same as Table 1 in the main text. The *p*-values correspond to the maximum or minimum TFCE and are based on permutation test (two-tailed) using TFCE as the test statistic. Source data are provided as a Source Data file.

Model	Regressor	Cluster size	Start time (ms)	End time (ms)	Maximum or minimum TFCE	*p*-value	Peak time (ms)
GLM-1	Current SV	84	440	1270	16050565	<0.0001	770
	Previous SV	113	380	1500	-10108427	<0.0001	1030
GLM-2	Current SV	83	450	1270	12276075	<0.0001	800
	Previous SV	113	380	1500	-10478213	<0.0001	1020
GLM-3	Current SV	130	210	1500	13170589	<0.0001	790
	Previous SV	121	140	1340	-7699084	<0.0001	830
GLM-4	Current SV	82	450	1260	12923489	<0.0001	800
	Previous SV	112	390	1500	-9882367	<0.0001	1020
GLM-5	Current SV	84	440	1270	14950391	<0.0001	760
	Previous SV	112	390	1500	-10644560	<0.0001	1020
GLM-6	Current SV	6	-120	-70	-307970	0.0487	-80
		85	430	1270	13917136	<0.0001	770
	Previous SV	113	380	1500	-7624380	<0.0001	1020
	Power from previous trial	19	-600	-420	-204042	0.0202	-460
GLM-1 ITI = 1 s	Current SV	11	-960	-860	378495	0.0366	-910
		108	430	1500	4520680	<0.0001	800
	Previous SV	12	220	330	-312217	0.0419	290
GLM-1 ITI = 1.5 s	Previous SV	108	430	1500	-3321347	<0.0001	1280
GLM-1 ITI = 2 s	Current SV	7	-110	-50	-278142	0.0435	-70
		99	170	1150	3599589	<0.0001	790
	Previous SV	116	350	1500	-5418119	<0.0001	1050

Supplementary Table 7: OFC beta (13-30 Hz), alpha (8-12 Hz), and theta (4-7 Hz) activity. Summary of statistical analysis examining the effects of current and previous subjective value. Conventions are the same as Table 1 in the main text. The *p*-values correspond to the maximum or minimum TFCE and are based on permutation test (two-tailed) using TFCE as the test statistic. Source data are provided as a Source Data file.

Frequency band	Regressor	Cluster size	Start time (ms)	End time (ms)	Maximum or minimum TFCE	*p*-value	Peak time (ms)
Beta	Current SV	16	-940	-790	439425	0.0285	-860
		12	-520	-410	-452762	0.0258	-460
	Previous SV	36	-780	-430	660197	0.0133	-580
		18	840	1010	385429	0.039	930
Alpha	Current SV	17	-470	-310	-449951	0.0259	-360
		22	-190	20	471555	0.0195	-140
		58	540	1110	-3227908	<0.0001	830
	Previous SV	20	-480	-290	570823	0.0158	-370
		102	490	1500	18137892	<0.0001	960
Theta	Current SV	5	-400	-360	-417332	0.0497	-380
		8	-150	-80	475021	0.0435	-110
		93	580	1500	-19634679	<0.0001	960
	Previous SV	28	-550	-280	692177	0.0261	-510
		110	490	1500	43945586	<0.0001	1340

Supplementary Table 8: OFC activity in the time-frequency space. Summary of statistical analysis examining the effects of current and previous subjective value. Conventions are the same as Table 1 in the main text. The *p*-values correspond to the maximum or minimum TFCE and are based on permutation test (two-tailed) using TFCE as the test statistic. Source data are provided as a Source Data file.

Regressor	Cluster size	Start time (ms)	End time (ms)	Frequency range (Hz)	Maximum or minimum TFCE	*p*-value	Peak time (ms)	Peak frequency (Hz)
Current SV	4547	-70	1500	(22, 150)	1814219	<0.001	760	84
	460	560	1500	(4, 24)	-593651	<0.001	850	6
	140	1180	1500	(132, 150)	109157	0.015	1460	140
	117	-140	210	(32, 46)	-122188	0.019	-20	42
	50	-490	-290	(6, 12)	-119255	0.02	-330	6
	15	-510	-420	(14, 16)	-105159	0.037	-440	16
	8	510	580	(22, 22)	-96499	0.049	550	22
	8	540	590	(10, 12)	-106870	0.035	570	12
	3	1360	1380	(132, 132)	90233	0.041	1370	132
	2	1060	1070	(130, 130)	104626	0.019	1060	130
	1	410	410	(58, 58)	89793	0.042	410	58
	1	1210	1210	(62, 62)	124038	0.007	1210	62
	1	1260	1260	(96, 96)	131017	0.006	1260	96
Previous SV	3942	-40	1500	(20, 150)	-755778	<0.001	970	56
	859	230	1500	(4, 24)	1292569	<0.001	1360	4
	13	1420	1500	(90, 94)	-90208	0.043	1470	92
	5	1340	1380	(104, 104)	-96185	0.03	1340	104
	4	440	470	(128, 128)	-107898	0.022	460	128
	2	210	220	(48, 48)	-90484	0.042	220	48
	1	1100	1100	(132, 132)	-93380	0.037	1100	132

Supplementary Table 9: Orbitofrontal cortex (OFC). MNI coordinates of the 166 OFC contacts. Source data are provided as a Source Data file.

No	Subject	X	Y	Z	Region	No	Subject	X	Y	Z	Region
1	1	12	21	-20	mOFC	49	7	-12	19	-16	mOFC
2	1	16	22	-18	mOFC	50	7	-17	21	-13	mOFC
3	1	20	24	-18	mOFC	51	7	-22	23	-11	cOFC
4	1	29	27	-14	cOFC	52	7	-27	26	-9	cOFC
5	1	40	26	4	lOFC	53	7	-33	28	-6	cOFC
6	1	45	27	3	lOFC	54	7	-38	29	-4	lOFC
7	1	50	28	2	lOFC	55	7	-42	30	-3	lOFC
8	1	54	28	2	lOFC	56	7	-49	31	0	lOFC
9	2	-11	21	-19	mOFC	57	7	-51	33	2	lOFC
10	2	-15	22	-16	mOFC	58	8	-12	21	-14	mOFC
11	2	-19	22	-13	mOFC	59	8	-17	22	-12	mOFC
12	3	31	34	-1	cOFC	60	8	-28	26	-8	cOFC
13	3	35	34	-1	cOFC	61	8	-31	28	-7	cOFC
14	3	40	35	0	lOFC	62	8	-36	30	-5	cOFC
15	3	44	36	0	lOFC	63	8	-40	31	-3	lOFC
16	3	21	27	-17	mOFC	64	9	29	34	-1	cOFC
17	3	24	27	-12	cOFC	65	9	35	35	1	cOFC
18	3	26	27	-8	cOFC	66	9	40	35	1	lOFC
19	3	29	28	-3	cOFC	67	9	15	24	-12	mOFC
20	3	33	29	6	cOFC	68	9	20	27	-12	mOFC
21	4	10	23	-23	mOFC	69	9	25	29	-12	cOFC
22	4	16	24	-22	mOFC	70	9	29	32	-12	cOFC
23	4	20	24	-21	mOFC	71	9	35	34	-13	cOFC
24	4	26	26	-20	cOFC	72	9	39	36	-13	cOFC
25	4	32	26	-19	cOFC	73	9	10	27	-21	mOFC
26	4	37	27	-19	cOFC	74	9	11	30	-18	mOFC
27	4	34	36	-17	cOFC	75	9	13	33	-14	mOFC
28	4	34	36	-11	cOFC	76	10	-11	26	-15	mOFC
29	4	35	36	-5	cOFC	77	10	-16	27	-13	mOFC
30	4	35	36	0	cOFC	78	10	-21	29	-11	mOFC
31	4	37	36	1	cOFC	79	10	-24	31	-10	cOFC
32	4	13	37	-22	mOFC	80	10	-29	34	-9	cOFC
33	5	-30	32	4	cOFC	81	10	-34	35	-8	cOFC
34	5	-35	32	4	cOFC	82	10	-38	38	-7	lOFC
35	5	-40	32	4	lOFC	83	10	-41	21	7	lOFC
36	5	-13	21	-14	mOFC	84	11	9	24	-19	mOFC
37	5	-24	28	-4	cOFC	85	11	13	27	-16	mOFC
38	5	-27	30	-1	cOFC	86	11	17	29	-13	mOFC
39	5	-30	32	2	cOFC	87	11	21	32	-9	cOFC
40	5	-35	35	6	cOFC	88	11	24	34	-6	cOFC
41	6	-15	20	-12	mOFC	89	11	-9	19	-20	mOFC
42	6	-28	27	-6	cOFC	90	11	-12	22	-17	mOFC
43	6	-31	29	-5	cOFC	91	11	-17	25	-13	mOFC
44	6	-35	31	-3	cOFC	92	11	-21	28	-10	mOFC
45	6	-40	32	-1	lOFC	93	11	-24	31	-6	cOFC
46	6	-45	35	1	lOFC	94	11	-28	33	-3	cOFC
47	6	-15	23	-19	mOFC	95	11	-31	36	1	cOFC
48	6	-15	26	-15	mOFC	96	12	-25	31	7	cOFC

No	Subject	X	Y	Z	Region
97	13	-11	15	-25	mOFC
98	13	-15	16	-23	mOFC
99	13	-19	18	-20	mOFC
100	13	-24	20	-15	cOFC
101	13	-27	22	-13	cOFC
102	13	-31	23	-10	cOFC
103	13	-36	25	-6	cOFC
104	13	-40	26	-4	lOFC
105	13	-44	28	-2	lOFC
106	13	-48	30	2	lOFC
107	14	10	28	-21	mOFC
108	14	15	28	-18	mOFC
109	14	19	28	-16	mOFC
110	14	24	29	-13	cOFC
111	14	28	29	-11	cOFC
112	14	32	31	-8	cOFC
113	14	36	32	-6	cOFC
114	14	41	35	-3	lOFC
115	14	45	36	-2	lOFC
116	15	-8	38	-23	mOFC
117	15	-12	41	-19	mOFC
118	15	-17	42	-16	mOFC
119	15	-19	29	-10	mOFC
120	15	-23	31	-8	cOFC
121	15	-27	32	-7	cOFC
122	15	-32	33	-6	cOFC
123	15	-38	34	-4	lOFC
124	15	-42	36	-3	lOFC
125	15	-45	39	-1	lOFC
126	16	-11	23	-18	mOFC
127	16	-17	25	-14	mOFC
128	16	-21	27	-12	mOFC
129	16	-25	29	-10	cOFC
130	16	-30	31	-8	cOFC
131	16	-34	32	-6	cOFC
132	16	-40	33	-4	lOFC
133	17	-9	23	-22	mOFC
134	17	-14	25	-21	mOFC
135	17	-19	27	-19	mOFC
136	17	-23	29	-18	cOFC
137	17	-29	31	-16	cOFC
138	17	-33	34	-15	cOFC
139	17	-38	35	-14	lOFC
140	17	-43	37	-13	lOFC
141	17	-48	40	-12	lOFC
142	17	-53	42	-11	lOFC
143	17	-38	23	10	lOFC
144	17	-25	29	8	cOFC
145	18	12	14	-21	mOFC
146	18	17	16	-20	mOFC
147	18	21	18	-18	cOFC
148	18	26	20	-16	cOFC
149	18	30	22	-14	cOFC
150	18	34	25	-12	cOFC
151	18	38	26	-11	cOFC
152	18	43	28	-9	lOFC
153	18	46	30	-8	lOFC
154	18	50	32	-6	lOFC
155	19	8	23	-24	mOFC
156	19	12	25	-23	mOFC
157	19	16	28	-20	mOFC
158	19	20	30	-18	mOFC
159	19	24	33	-16	cOFC
160	19	29	36	-14	cOFC
161	19	33	39	-12	cOFC
162	19	36	41	-11	cOFC
163	20	7	28	-26	mOFC
164	20	9	30	-22	mOFC
165	20	13	32	-17	mOFC
166	20	14	34	-14	mOFC

Supplementary Table 10: Amygdala. MNI coordinates of the 30 amygdala contacts. Source data are provided as a Source Data file.

No	Subject	X	Y	Z
1	1	23	-2	-22
2	1	27	0	-22
3	1	32	2	-23
4	2	-25	-4	-21
5	2	-29	-3	-20
6	2	-34	-2	-20
7	4	23	-4	-27
8	4	28	-2	-26
9	4	33	-1	-26
10	5	-27	-6	-23
11	5	-32	-4	-22
12	11	26	-4	-23
13	11	31	-3	-23
14	11	-21	-5	-24
15	11	-27	-4	-24
16	11	-31	-4	-24
17	13	-35	0	-18
18	13	-28	-4	-25
19	13	-33	-3	-25
20	15	-33	-6	-25
21	16	-30	-4	-25
22	17	-18	-5	-24
23	17	-23	-4	-25
24	17	-29	-3	-25
25	19	17	-1	-29
26	19	22	-1	-28
27	19	26	0	-27
28	19	30	1	-27
29	20	29	-5	-23
30	20	34	-4	-23

Supplementary Table 11: Hippocampus. MNI coordinates of 126 hippocampus contacts. Source data are provided as a Source Data file.

No	Subject	X	Y	Z
1	1	25	-16	-20
2	1	30	-15	-20
3	1	35	-14	-20
4	1	39	-13	-20
5	1	22	-28	-14
6	1	31	-28	-14
7	1	36	-28	-15
8	2	-32	-18	-20
9	2	-37	-17	-19
10	2	-30	-32	-7
11	2	-35	-33	-8
12	4	27	-15	-18
13	4	32	-14	-17
14	4	38	-14	-17
15	4	23	-31	-9
16	4	28	-33	-9
17	4	32	-34	-8
18	4	38	-34	-7
19	5	-18	-9	-24
20	5	-23	-8	-23
21	5	-21	-17	-21
22	5	-26	-17	-21
23	5	-31	-17	-21
24	5	-35	-16	-21
25	5	-26	-30	-6
26	5	-30	-31	-6
27	5	-35	-32	-6
28	6	-20	-12	-24
29	6	-25	-12	-24
30	6	-30	-12	-24
31	6	-35	-14	-23
32	6	-23	-27	-16
33	6	-28	-27	-15
34	6	-33	-26	-14
35	6	-38	-27	-13
36	7	-26	-38	-13
37	8	-19	-10	-23
38	8	-24	-9	-23
39	8	-28	-8	-24
40	8	-33	-8	-25
41	8	-22	-17	-19
42	8	-27	-18	-18
43	8	-31	-20	-18
44	8	-24	-35	-14
45	8	-29	-35	-13
46	8	-34	-35	-12
47	10	-23	-15	-22
48	10	-29	-15	-20
49	10	-32	-15	-20
50	10	-38	-15	-19
51	11	21	-7	-22
52	11	26	-17	-22
53	11	29	-16	-21
54	11	35	-15	-18
55	11	23	-37	-7
56	11	28	-37	-7
57	11	33	-36	-7
58	11	38	-35	-6
59	11	-23	-18	-18
60	11	-28	-18	-18
61	11	-33	-19	-18
62	11	-20	-33	-11
63	11	-24	-34	-11
64	11	-28	-36	-10
65	11	-34	-37	-9
66	12	-24	-11	-20
67	12	-28	-11	-20
68	12	-33	-10	-21
69	12	-23	-20	-17
70	12	-27	-20	-18
71	12	-31	-20	-17
72	12	-36	-20	-16
73	12	-20	-30	-16
74	12	-25	-30	-15
75	12	-29	-30	-14
76	12	-33	-31	-12
77	12	-37	-30	-12
78	12	20	-17	-21
79	12	25	-15	-21
80	12	29	-13	-21
81	12	33	-12	-22
82	12	37	-10	-22
83	12	30	-24	-23
84	13	-35	-18	-23
85	15	-23	-7	-25
86	15	-28	-7	-25
87	15	-22	-18	-18
88	15	-26	-18	-17
89	15	-31	-18	-17
90	15	-36	-19	-17
91	16	-20	-7	-26
92	16	-25	-6	-26
93	16	-24	-18	-21
94	16	-29	-18	-20
95	16	-34	-17	-20
96	16	-23	-30	-15
97	16	-27	-31	-14
98	16	-33	-32	-13
99	16	-37	-31	-13
100	17	-34	-2	-25
101	18	23	-7	-29
102	18	28	-6	-29
103	18	33	-5	-29
104	18	26	-17	-21
105	18	31	-17	-21
106	18	35	-16	-21
107	18	40	-15	-21
108	18	31	-33	-15
109	18	35	-33	-13
110	19	21	-12	-27
111	19	25	-12	-27
112	19	30	-12	-25
113	19	35	-12	-24
114	19	24	-28	-17
115	19	29	-28	-17
116	19	34	-28	-18
117	20	18	-6	-24
118	20	24	-6	-23
119	20	24	-17	-20
120	20	28	-17	-20
121	20	33	-16	-20
122	20	38	-16	-19
123	20	23	-24	-16
124	20	28	-24	-15
125	20	33	-23	-14
126	20	38	-24	-14

Supplementary Table 12: Striatum. MNI coordinates of the 25 striatum contacts. Source data are provided as a Source Data file.

No	Subject	X	Y	Z
1	1	33	-18	-1
2	2	-32	3	-8
3	2	-31	5	-3
4	2	-30	6	2
5	2	-29	8	8
6	4	23	16	6
7	5	-33	3	-4
8	5	-32	6	1
9	5	-32	-11	-5
10	5	-31	-14	0
11	5	-30	-18	5
12	5	-30	-21	9
13	7	-31	-2	-2
14	7	-31	0	2
15	7	-29	4	8
16	11	31	10	0
17	11	29	13	6
18	12	-11	15	-12
19	12	-14	19	-8
20	12	-17	22	-4
21	12	-20	25	0
22	12	-33	-14	1
23	12	-31	-17	8
24	16	-34	-10	-2
25	16	-32	-15	5

Supplementary Table 13: Insula. MNI coordinates of the 169 insula contacts. Source data are provided as a Source Data file.

No	Subject	X	Y	Z	No	Subject	X	Y	Z	No	Subject	X	Y	Z
1	1	36	4	-23	49	5	-30	-29	19	97	12	-34	2	14
2	1	38	-18	-2	50	5	-30	-33	23	98	12	-36	-10	-7
3	1	42	-18	-2	51	7	-31	-14	19	99	12	-29	-22	16
4	1	30	24	5	52	7	-36	-14	19	100	13	-36	4	3
5	1	39	2	-9	53	7	-30	7	12	101	13	-40	4	5
6	1	38	6	-4	54	7	-28	10	18	102	13	-36	4	3
7	1	37	8	0	55	8	-33	10	1	103	13	-40	4	5
8	1	36	10	5	56	8	-31	12	6	104	13	-33	6	-11
9	1	35	12	11	57	8	-31	14	10	105	13	-33	11	-6
10	1	38	-8	8	58	8	-30	15	15	106	13	-31	17	0
11	1	37	-10	13	59	8	-37	-6	-8	107	13	-30	23	6
12	1	36	-13	19	60	8	-36	-10	-2	108	13	-32	-23	19
13	2	-30	7	18	61	8	-35	-13	2	109	13	-37	-23	20
14	2	-39	-1	-19	62	8	-35	-15	6	110	13	-37	-2	-8
15	2	-32	-28	6	63	8	-34	-19	11	111	13	-35	-6	-1
16	2	-38	-8	-12	64	8	-34	-22	15	112	13	-34	-11	6
17	2	-44	-8	-11	65	8	-32	-25	20	113	13	-33	-16	13
18	2	-28	10	13	66	8	-32	-27	24	114	13	-31	-22	19
19	2	-27	12	18	67	9	36	5	-9	115	14	30	14	5
20	2	-42	-6	-5	68	9	35	9	-4	116	14	34	15	7
21	2	-41	-8	0	69	9	32	12	1	117	14	38	15	8
22	2	-40	-11	4	70	9	31	15	4	118	14	31	-21	20
23	2	-39	-14	9	71	9	30	19	9	119	14	36	-23	20
24	2	-38	-16	14	72	9	28	23	14	120	14	-37	-16	16
25	2	-37	-18	18	73	10	-34	-12	20	121	14	-42	-16	16
26	2	-35	-21	23	74	10	-27	14	9	122	15	-35	-16	14
27	3	37	8	2	75	10	-32	16	8	123	15	-40	-17	15
28	3	36	11	6	76	10	-37	18	7	124	15	-30	12	9
29	3	35	14	10	77	10	-32	10	-5	125	15	-36	12	12
30	3	34	17	14	78	10	-31	13	-1	126	15	-37	8	-10
31	3	31	28	2	79	10	-31	16	2	127	15	-35	11	-6
32	4	29	17	7	80	10	-29	20	7	128	15	-34	14	-3
33	4	34	18	8	81	10	-28	22	11	129	15	-33	18	2
34	4	38	18	8	82	11	40	-23	-3	130	15	-32	21	5
35	4	36	-22	2	83	11	33	7	-6	131	15	-30	26	9
36	4	41	-22	2	84	11	38	3	-7	132	15	-39	-17	3
37	4	40	1	-7	85	11	37	0	1	133	15	-44	-17	3
38	4	38	5	-1	86	11	35	-4	9	134	16	-35	4	5
39	4	38	9	3	87	11	35	-7	17	135	16	-39	4	7
40	4	37	12	7	88	11	-35	1	-16	136	16	-32	-21	16
41	4	37	16	11	89	11	-35	6	-10	137	16	-37	-19	18
42	5	-36	-10	15	90	11	-34	12	-4	138	16	-36	-21	-5
43	5	-41	-10	15	91	11	-33	16	2	139	16	-40	-22	-4
44	5	-34	1	-8	92	11	-31	22	9	140	16	-30	-21	13
45	5	-31	10	6	93	12	-37	-4	-8	141	16	-29	-27	20
46	5	-30	12	10	94	12	-36	-3	-3	142	17	-29	22	9
47	5	-29	16	15	95	12	-35	0	3	143	17	-33	22	9
48	5	-30	-26	15	96	12	-35	1	9	144	17	-35	9	-10

No	Subject	X	Y	Z
145	17	-33	13	-6
146	17	-31	16	-4
147	17	-29	22	1
148	17	-28	25	4
149	17	-31	0	17
150	18	37	-10	15
151	18	41	-10	17
152	18	43	-5	-12
153	18	43	-9	-6
154	18	41	-13	3
155	18	39	-16	11
156	18	39	-20	18
157	18	39	-23	-5
158	19	37	-21	4
159	19	36	16	-12
160	19	35	18	-7
161	19	34	20	-2
162	19	32	22	2
163	19	31	25	8
164	20	33	-26	5
165	20	34	5	-12
166	20	32	10	-7
167	20	31	16	-3
168	20	29	21	1
169	20	28	27	7

Supplementary Table 14: Anterior cingulate cortex (ACC) and midcingulate cortex (MCC). MNI coordinates of the 81 ACC and MCC contacts. Source data are provided as a Source Data file.

No	Subject	X	Y	Z	No	Subject	X	Y	Z
1	2	-6	35	15	49	13	-8	38	9
2	2	-10	35	15	50	13	-12	39	10
3	2	-15	35	17	51	13	-16	42	13
4	2	-10	13	29	52	13	-7	13	38
5	2	-14	14	29	53	13	-12	15	39
6	3	11	33	-4	54	14	7	46	14
7	3	7	26	18	55	14	10	49	17
8	3	12	26	18	56	14	11	13	26
9	3	9	20	38	57	14	12	-14	35
10	3	10	-5	47	58	14	5	38	-6
11	3	15	-5	46	59	14	9	38	-4
12	3	10	30	-1	60	14	15	39	0
13	3	13	33	16	61	14	-9	29	25
14	3	13	34	22	62	14	-14	31	26
15	3	14	35	27	63	14	-8	11	40
16	4	8	45	6	64	14	-11	13	42
17	4	13	45	7	65	15	-3	38	6
18	4	18	46	8	66	15	-8	39	10
19	4	10	33	19	67	15	-11	40	12
20	4	16	33	20	68	15	-16	42	15
21	4	10	10	31	69	15	-5	33	29
22	4	17	42	1	70	15	-9	35	30
23	4	17	43	6	71	15	-4	16	37
24	5	-6	30	33	72	15	-9	16	40
25	5	-4	33	7	73	15	-5	-2	41
26	5	-5	8	30	74	15	-10	-3	42
27	5	-10	8	30	75	17	-3	44	26
28	7	-12	43	13	76	17	-4	16	41
29	7	-15	43	17	77	17	-9	17	44
30	7	-11	36	-2	78	17	-9	4	32
31	7	-14	37	0	79	17	-7	35	9
32	7	-10	23	23	80	17	-13	37	11
33	7	-5	-4	37	81	17	-17	39	12
34	7	-12	-4	37					
35	7	-6	4	44					
36	7	-11	5	45					
37	8	-8	35	0					
38	9	8	33	-5					
39	9	8	38	31					
40	9	14	23	23					
41	10	-7	-2	39					
42	10	-12	-2	39					
43	10	-5	18	42					
44	10	-7	35	-4					
45	10	-12	36	-2					
46	10	-8	27	15					
47	10	-12	28	17					
48	13	-3	36	6					

Supplementary Table 15: Posterior cingulate cortex (PCC). MNI coordinates of 31 PCC contacts. Source data are provided as a Source Data file.

No	Subject	X	Y	Z
1	1	14	-44	29
2	3	11	-46	41
3	3	16	-45	42
4	9	9	-33	53
5	9	9	-46	28
6	9	14	-46	27
7	11	23	-37	-7
8	12	-15	-52	-6
9	12	-1	-39	35
10	12	-5	-38	36
11	12	-10	-38	37
12	12	-16	-38	39
13	12	-19	-38	40
14	14	8	-15	34
15	14	17	-15	36
16	14	8	-38	27
17	16	-6	-35	33
18	16	-11	-35	34
19	16	-16	-36	34
20	16	-12	-61	5
21	16	-17	-64	6
22	16	-22	-65	7
23	16	-7	-49	39
24	16	-10	-49	43
25	18	10	-48	13
26	18	14	-49	14
27	18	10	-48	13
28	18	14	-49	14
29	18	6	-42	42
30	18	11	-44	43
31	18	16	-45	43

Supplementary Table 16: Intraparietal sulcus (IPS). MNI coordinates of the 62 IPS contacts. Source data are provided as a Source Data file.

No	Subject	X	Y	Z	No	Subject	X	Y	Z
1	1	38	-25	40	49	18	42	-54	23
2	1	42	-25	39	50	18	46	-54	24
3	1	31	-49	57	51	18	51	-55	25
4	1	35	-49	56	52	18	29	-49	45
5	1	40	-49	56	53	18	34	-50	46
6	1	44	-49	55	54	18	39	-51	46
7	1	33	-44	26	55	18	44	-52	46
8	1	37	-44	25	56	18	48	-53	46
9	1	33	-26	42	57	20	34	-32	34
10	1	33	-29	48	58	20	39	-32	34
11	2	-38	-46	23	59	20	44	-32	35
12	2	-33	-30	37	60	20	49	-33	36
13	3	30	-45	45	61	20	53	-33	37
14	3	35	-44	46	62	20	58	-32	38
15	3	39	-44	47					
16	3	44	-45	47					
17	5	-29	-40	33					
18	5	-29	-44	38					
19	9	30	-34	50					
20	9	41	-36	49					
21	9	46	-35	50					
22	12	-37	-36	46					
23	12	-41	-36	47					
24	12	-24	-38	42					
25	13	-29	-38	39					
26	13	-28	-42	45					
27	13	-27	-44	52					
28	14	32	-37	34					
29	14	36	-38	35					
30	14	40	-37	36					
31	14	45	-37	36					
32	14	50	-37	37					
33	16	-30	-37	33					
34	16	-36	-37	32					
35	16	-41	-38	32					
36	16	-46	-38	32					
37	16	-51	-38	33					
38	16	-14	-50	47					
39	16	-18	-50	51					
40	16	-24	-44	42					
41	16	-24	-50	49					
42	16	-24	-55	57					
43	18	42	-54	23					
44	18	46	-54	24					
45	18	51	-55	25					
46	18	35	-33	39					
47	18	34	-38	46					
48	18	32	-42	53					
