# Supplementary material for: Electrophysiological population dynamics reveal context dependencies during decision making in human frontal cortex
Source: Nat Commun. 2023 Nov 28;14:7821. doi: 10.1038/s41467-023-42092-x (PMC10684521; doi:10.1038/s41467-023-42092-x)
Supplement: Supplementary file 3 — Reporting Summary [file 41467_2023_42092_MOESM3_ESM.pdf]

nature portfolio

Corresponding author(s): Wan-Yu Shih, Paul W. Glimcher, Shih-Wei Wu

Last updated by author(s): Aug 12, 2023

Reporting Summary

Nature Portfolio wishes to improve the reproducibility of the work that we publish. This form provides structure for consistency and transparency in reporting. For further information on Nature Portfolio policies, see our Editorial Policies and the Editorial Policy Checklist.

Statistics

For all statistical analyses, confirm that the following items are present in the figure legend, table legend, main text, or Methods section.

n/a	Confirmed	
☐	☒	The exact sample size (*n*) for each experimental group/condition, given as a discrete number and unit of measurement
☐	☒	A statement on whether measurements were taken from distinct samples or whether the same sample was measured repeatedly
☐	☒	The statistical test(s) used AND whether they are one- or two-sided<br>*Only common tests should be described solely by name; describe more complex techniques in the Methods section.*
☐	☒	A description of all covariates tested
☐	☒	A description of any assumptions or corrections, such as tests of normality and adjustment for multiple comparisons
☐	☒	A full description of the statistical parameters including central tendency (e.g. means) or other basic estimates (e.g. regression coefficient) AND variation (e.g. standard deviation) or associated estimates of uncertainty (e.g. confidence intervals)
☐	☒	For null hypothesis testing, the test statistic (e.g. *F*, *t*, *r*) with confidence intervals, effect sizes, degrees of freedom and *P* value noted<br>*Give P values as exact values whenever suitable.*
☒	☐	For Bayesian analysis, information on the choice of priors and Markov chain Monte Carlo settings
☒	☐	For hierarchical and complex designs, identification of the appropriate level for tests and full reporting of outcomes
☐	☒	Estimates of effect sizes (e.g. Cohen's *d*, Pearson's *r*), indicating how they were calculated

Our web collection on statistics for biologists contains articles on many of the points above.

Software and code

Policy information about availability of computer code

Data collection	MATLAB R2018 to R2021, Psychophysics Toolbox Version 3
Data analysis	MATLAB R2018 to R2021, EEGLAB (version 2022.1), ERPLAB (version 9.00), FSL version 6.0, SPM12, https://github.com/fahsuanlin/fhlin_toolbox. The custom computer code used for the analyses reported in this study are available at Open Science Framework (https://osf.io/f92yv/?view_only=95befcd791b9429692fc804a2876918a).

For manuscripts utilizing custom algorithms or software that are central to the research but not yet described in published literature, software must be made available to editors and reviewers. We strongly encourage code deposition in a community repository (e.g. GitHub). See the Nature Portfolio guidelines for submitting code & software for further information.

Data

Policy information about availability of data

All manuscripts must include a data availability statement. This statement should provide the following information, where applicable:

- Accession codes, unique identifiers, or web links for publicly available datasets
- A description of any restrictions on data availability
- For clinical datasets or third party data, please ensure that the statement adheres to our policy

Source data and statistics used to make each figure are provided with this paper. The high-gamma activity data generated in this study have been deposited in Open

Science Framework (https://osf.io/f92yv/?view_only=95befcd791b9429692fc804a2876918a). The raw data analyzed in this study are available upon request made to the corresponding authors.

Human research participants

Policy information about studies involving human research participants and Sex and Gender in Research.

Reporting on sex and gender	The findings do not apply to only one sex or gender; the sex and gender were not considered in study design; sex and/or gender was determined based on self-reporting.
Population characteristics	age 16 to 51; diagnosis: drug-resistant epilepsy; normal controls: age 20 to 27
Recruitment	Participants were drug-resistant epilepsy patients who were treated for epilepsy. As part of the treatment plan, stereo electroencephalography (sEEG) data were obtained in order to identify potential epileptogenic zone. We recruited them through the Epilepsy Surgery team at the Taipei Veterans General Hospital responsible for treating these patients. The patients were recruited when the treating physician team agreed that the patients were in good health form to perform the behavioral task. We also collected behavioral data from 35 healthy subjects in the normal population as normal control. The healthy subjects were recruited from the Internet (PTT Bulletin Board System, ptt.cc). For both the patients and healthy subjects, we are not aware of any self-selection bias during the recruitment process.
Ethics oversight	For the epilepsy patients, the study was approved by the Taipei Veterans General Hospital Institutional Review Board; For the healthy normal control subjects, the study was approved by the National Yang Ming Chiao Tung University Institutional Review Board.

Note that full information on the approval of the study protocol must also be provided in the manuscript.

Field-specific reporting

Please select the one below that is the best fit for your research. If you are not sure, read the appropriate sections before making your selection.

☒ Life sciences ☐ Behavioural & social sciences ☐ Ecological, evolutionary & environmental sciences

For a reference copy of the document with all sections, see nature.com/documents/nr-reporting-summary-flat.pdf

Life sciences study design

All studies must disclose on these points even when the disclosure is negative.

Sample size	The sample size chosen in this study (20 participants) was based on prior experience and common research standards in the literature on human cognition research. Based on previous studies related to our study, which is in human decision making, a sample size of 16 or more participants is generally recommended and widely seen in previous studies, as far as obtaining robust statistical conclusions regarding the choice behavior of the particiapnts (Tom et al., 2007). For the neural side of this study, which used sEEG, sample size varies a lot in previous studies, from 6 to 30 plus participants (Domenech et al., 2020; Saez et al., 2018; Lopez-Persem et al., 2020). We chose our sample size so that it does not deviate much from the mean of this distributions, and would allow us to investigate individual differences in both neural activity and behavior. For the healthy controls, we had 35 participants. They only performed the behavioral auction task. The purpose of collecting their data is to compare the decision-making behavior of the patients with these normal subjects. In our experience and previous publications regarding this behavioral task, the sample size was generally around 20 (Plassmann et al., 2007). We therefore believe 35 is a good number to understand the behavioral aspect of the auction task.
Data exclusions	1. Trials where the response time (RT) was 3 standard deviation away from the mean RT of the subject were excluded from further analysis (usually less than 1% of trials), as they could very well indicate disruptions of the experiment outside of the experimenters' control (e.g., visits from clinicians, nurses, and/or staffs).<br>2. Trial epochs with interictal spikes were identified through visual inspection and were excluded from further data analysis.
Replication	We are currently collecting new data from different patients performing the same experimental task as this study as part of a new study. Once we reach the same or similar sample size as this study, we will analyze the part of neural data where the participants performed the same task, as our attempt to replicate this findings.
Randomization	We did not have different experimental groups in this study and therefore there is no need for randomization.
Blinding	The study did not have different experimental groups so it is not necessary to be blinded by randomization/allocation.

Reporting for specific materials, systems and methods

We require information from authors about some types of materials, experimental systems and methods used in many studies. Here, indicate whether each material, system or method listed is relevant to your study. If you are not sure if a list item applies to your research, read the appropriate section before selecting a response.

Materials & experimental systems

n/a	Involved in the study
☒	☐ Antibodies
☒	☐ Eukaryotic cell lines
☒	☐ Palaeontology and archaeology
☒	☐ Animals and other organisms
☒	☐ Clinical data
☒	☐ Dual use research of concern

Methods

n/a	Involved in the study
☒	☐ ChIP-seq
☒	☐ Flow cytometry
☐	☒ MRI-based neuroimaging

Magnetic resonance imaging

Experimental design

Design type	T1-weighted structural scan
Design specifications	N/A
Behavioral performance measures	N/A

Acquisition

Imaging type(s)	structural
Field strength	1.5
Sequence & imaging parameters	For each patient, T1-weighed structural MRI images were collected on a 1.5T Signa HDxt scanner (GE Healthcare, Milwaukee, WI, USA) before and after the surgery for electrode implantation. The MR images were taken along the axial plane using a fast spoiled gradient-recalled echo sequence (axial slice thickness=1 mm; TR=10.02 ms, TE=4.28 ms, TI=0 ms, flip angle=15°, bandwidth=31.2 kHz, matrix=256×256, FOV=256×256 mm).
Area of acquisition	Whole brain

Diffusion MRI ☐ Used ☒ Not used

Preprocessing

Preprocessing software	N/A
Normalization	To identify the anatomical location of electrode contacts across different subjects, we transformed the electrode contact location from the subject's native space to standard Montreal Neurological Institute (MNI) space. To do that, we used three sets of brain images collected from each subject: the T1-weighted image prior to electrode implementation (pre-T1), the T1-weighted image after electrode implementation (post-T1), and the CT image after electrode implantation (post-CT). Our goal was to transform the CT image to MNI space. The reason we used the CT image to identify the electrode contact coordinates in the standard space was because the CT image, compared with T1-weighted image, suffers less distortion and therefore allows for more accurate mapping of the contact location. The transformation was performed using SPM12 (Wellcome Trust Centre for Neuroimaging, London, UK; https://www.fil.ion.ucl.ac.uk/spm/) and proceeded in the following three steps. First, the post-CT image was aligned to the post-T1 image with 4th degree B-Spline interpolation. Second, both the post-T1 image and the realigned post-CT image were aligned with the pre-T1 image, also with 4th degree B-Spline interpolation. Finally, the pre-T1, the realigned post-T1 and post-CT images were transformed to the standard MNI space (1 mm isotropic voxel size). The location of each contact in MNI space was obtained through the post-CT images in MNI space.
Normalization template	MNI152
Noise and artifact removal	N/A
Volume censoring	N/A

Statistical modeling & inference

Model type and settings	N/A
Effect(s) tested	N/A

Specify type of analysis: ☒ Whole brain ☐ ROI-based ☐ Both

Statistic type for inference (See Eklund et al. 2016)	N/A

Correction	N/A

Models & analysis

n/a	Involved in the study
☒	☐ Functional and/or effective connectivity
☒	☐ Graph analysis
☒	☐ Multivariate modeling or predictive analysis
